# Accelerated versus standard initiation of renal replacement therapy for critically ill patients with acute kidney injury: a systematic review and meta-analysis of RCT studies

**DOI:** 10.1186/s13054-020-03434-z

**Published:** 2021-01-05

**Authors:** Heng-Chih Pan, Ying-Ying Chen, I-Jung Tsai, Chih-Chung Shiao, Tao-Min Huang, Chieh-Kai Chan, Hung-Wei Liao, Tai-Shuan Lai, Yvonne Chueh, Vin-Cent Wu, Yung-Ming Chen

**Affiliations:** 1 Division of Nephrology, Department of Internal Medicine, Keelung Chang Gung Memorial Hospital, Keelung, Taiwan; 2 Graduate Institute of Clinical Medicine, College of Medicine, National Taiwan University, Taipei, Taiwan; 3 Chang Gung University College of Medicine, Taoyuan, Taiwan; 4 Community Medicine Research Center, Keelung Chang Gung Memorial Hospital, Keelung, Taiwan; 5 Division of Nephrology, Department of Internal Medicine, MacKay Memorial Hospital, Taipei, Taiwan; 6 Division of Nephrology, Department of Pediatrics, National Taiwan University Children’s Hospital, Taipei, Taiwan; 7 Division of Nephrology, Department of Internal Medicine, Saint Mary’s Hospital Luodong, Yilan, Taiwan; 8 Department of Internal Medicine, National Taiwan University Hospital, College of Medicine, National Taiwan University, 7 Chung-Shan South Road, Taipei, Taiwan; 9 Department of Internal Medicine, National Taiwan University Hospital, Hsin-Chu Branch, Hsin Chu County, Taiwan; 10 Chinru Clinic, Taipei, Taiwan; 11 Department of Family Medicine, Cleveland Clinic Akron General Hospital, Akron, OH USA; 12 National Taiwan University Hospital Study Group of ARF (NSARF), Taiwan Consortium for Acute Kidney Injury and Renal Diseases (CAKS, TCTC), Taipei, Taiwan

**Keywords:** Accelerated dialysis, Dialysis dependence, Free of dialysis, Mortality, Renal replacement therapy, Standard dialysis

## Abstract

**Background:**

Acute kidney injury (AKI) is a common yet possibly fatal complication among critically ill patients in intensive care units (ICU). Although renal replacement therapy (RRT) is an important supportive management for severe AKI patients, the optimal timing of RRT initiation for these patients is still unclear.

**Methods:**

In this systematic review, we searched all relevant randomized controlled trials (RCTs) that directly compared accelerated with standard initiation of RRT from PUBMED, MEDLINE, EMBASE, and Cnki.net published prior to July, 20, 2020. We extracted study characteristics and outcomes of being free of dialysis, dialysis dependence and mortality. We rated the certainty of evidence according to Cochrane methods and the GRADE approach.

**Results:**

We identified 56 published relevant studies from 1071 screened abstracts. Ten RCTs with 4753 critically ill AKI patients in intensive care unit (ICU) were included in this meta-analysis. In our study, accelerated and standard RRT group were not associated with all-cause mortality (log odds-ratio [OR]: − 0.04, 95% confidence intervals [CI] − 0.16 to 0.07, *p* = 0.46) and free of dialysis (log OR: − 0.03, 95% CI − 0.14 to 0.09, *p* = 0.65). In the subgroup analyses, accelerated RRT group was significantly associated with lower risk of all-cause mortality in the surgical ICU and for those who received continuous renal replacement therapy (CRRT). In addition, patients in these two subgroups had higher chances of being eventually dialysis-free. However, accelerated initiation of RRT augmented the risk of dialysis dependence in the subgroups of patients treated with non-CRRT modality and whose Sequential Organ Failure Assessment (SOFA) score were more than 11.

**Conclusions:**

In this meta-analysis, critically ill patients with severe AKI would benefit from accelerated RRT initiation regarding all-cause mortality and being eventually free of dialysis only if they were surgical ICU patients or if they underwent CRRT treatment. However, the risk of dialysis dependence was increased in the accelerated RRT group when those patients used non-CRRT modality or had high SOFA scores. All the literatures reviewed in this study were highly heterogeneous and potentially subject to biases.

*Trial registration* CRD42020201466, Sep 07, 2020. https://www.crd.york.ac.uk/prospero/display_record.php?RecordID=201466.

## Background

Acute kidney injury (AKI) is common in patients with critical illnesses admitted to intensive care units (ICUs) [1]. The most critical patients with kidney injury may need immediate renal replacement therapy (RRT; dialysis) since AKI is potentially accompanied with lethal complications, such as severe fluid overload, electrolyte disturbances, and acidemia [2]. However, when exactly to initiate RRT in the absence of compellingly lethal complications of AKI in critically ill patients remains unknown [3–5].

The question of whether to utilize accelerated or standard initiation of RRT has been long debated within the past two decades. Previously published related meta-analyses reported potential benefits of accelerated RRT in a subset of patients, but the conclusions have not been widely accepted due to the heterogeneity in the studies and limited number of patients in the randomized controlled trials (RCTs) [4, 6]. It had previously been concluded that early initialization of dialysis for AKI could be beneficial for surgical patients and in the setting of continuous renal replacement therapy (CRRT) [4]. However, initializing CRRT early has not shown a definitive benefit of patient survival and kidney recovery when compared to intermittent dialysis in other reports [7, 8].

Recently, the largest-to-date, multicenter RCT study focusing on this issue has been published, and it recruited patients globally [9]. Therefore, herein, we combined all the available RCT data and conducted a systematic review and meta-analysis to investigate whether accelerated or standard initiation of RRT in critically ill AKI patients is beneficial in terms of several outcomes, including mortality, free of dialysis, dialysis dependence and also scrutinize their subgroup analyses.

## Methods

### Search strategy and selection criteria

We reported the meta-analysis according to the Preferred Reporting Items of Systematic Reviews and Meta-Analyses (PRISMA) statement [10] and used Cochrane methods [11]. We prospectively submitted the systematic review protocol for registration on PROSPERO [CRD42020201466] (Additional file 1: Appendix 6).

### Data sources and search strategy

Electronic searches were performed on PubMed (Ovid), Medline, Embase, Cochrane library, and Cnki.net from inception to July 20, 2020. The search strategies are listed in Additional file 1: Appendix 1. We screened references by titles and abstracts and included related studies for further analysis. Reference lists of related studies, systematic reviews and meta-analyses were manually examined to identify any additional publications relevant to our analysis. Both abstracts and full papers were selected for quality assessment and data syntheses.

### Inclusion and exclusion methods

We enrolled RCT studies with the following inclusion criteria: (1) studies that clearly specified participants were randomized into either control or experiment group; in (2) literature search using MeSH terms or free-texts words with acute kidney injury, renal replacement therapy, as well as the words characterized with initiation; (3) participants included critical patients with AKI who were at least 18 years of age and were not previously on dialysis; (4) assessed at least one of these outcomes: free of RRT rate, in-hospital mortality, 28-day and 90-day mortality rates after hospital discharge, and dialysis dependence rate after hospital discharge. We excluded articles that did not clearly define the timing of RRT initiations, that included participants younger than 18 years of age, lacked outcomes aforementioned, and participant randomization was not clearly defined. Full-text papers were selected for quality assessment and data syntheses.

### Study selection and data extraction

Two investigators (Ying-Ying Chen and Heng-Chih Pan) independently reviewed the search results and identified eligible studies. Any resulting discrepancies were resolved by discussion with a third investigator (Chih-Chung Shiao). All relevant data were independently extracted from the included studies by two investigators (Ying-Ying Chen and Heng-Chih Pan) according to a standardized form. Extracted data included study characteristics (leading author, publication year, patient enrollment, sample size, events, duration of follow-up (weeks), the National Clinical Trial number) and participants’ baseline (age (years), gender (%), comorbidities, severity of the illness). When available, odds ratios and 95% confidence intervals (CIs) from the cohort or case-controlled studies were extracted. Other a priori determined parameters were the type of ICU setting (surgical /mixed or medical), criteria used for AKI and severe AKI diagnosis, cohort size, presence of sepsis, study quality, and the proportions of patients on mechanical ventilation. The baseline characteristics of included studies are illustrated in Table 1. The primary outcome was in-hospital mortality rates, while the secondary outcomes were free of RRT and RRT dependence. The survivors who did not need RRT at the end of the study were defined as being free of dialysis; others who were kept on dialysis were considered dialysis dependent. We also evaluated the 28-day and 90-day mortality rates after hospital discharge. Any disagreements were resolved by discussion with the investigator (Vin-Cent Wu).

**Table 1:** Characteristics of included comparative studies

References	Population setting and site	Nation, continence	Patients (early/late)	WWS*	Age	Male (%)	HTN/DM/HF/CKD (%)	Sepsis (%)	Definition of "Accelerated"	Definition of "Standard"	RRT modality	Primary outcome
Bouman et al. [16]	Multi-center mixed	Netherland	71 (35/36)	Yes	68.4	59.2	NA	N/A	Within 12 h after fulfilling the following criteria: UOP < 30 mL/h and CCr < 20 mL/min on 3-h sample	When the patient fulfilled the conventional criteria for RRT: BUN > 40 mmol/L, K > 6.5 mmol/L or severe pulmonary edema (CVP or pulmonary artery occlusion pressure of 16 mm Hg and lung edema with positive end expiratory pressure of 10 cm H_2_O and PO_2_/FIO_2_ ratio of 150 mm Hg	CRRT	In-hospital mortality, In-ICU mortality, 28-day mortality
Sugahara et al. [17]	Single-center, surgical	Japan	28 (14/14)	No	64.5	64.3	57.0/38.5/NA/NA	N/A	Within 12 h of UOP < 30 mL/h or < 750 mL/day	After 12 h of UOP < 20 mL/h or urine outpu*t* < 500 mL/day	CRRT	14-day mortality
Wald et al. [18]	Multi-center, mixed	Canada	100 (48/52)	Yes	63.1	72.0	53.0/39.0/11.0/NA	56.0	Within 12 h of fulfilling eligibility: at least two of the following: twofold increase in serum Cr from baseline; UOP < 6 mL/kg in the preceding 12 h; whole-blood NGAL > 400 ng/mL	Severe hyperkalemia (> 6 mmol/L); severe pulmonary edema; severe metabolic acidosis (serum bicarbonate < 10 mmol/L)	Mixed (IHD/CRRT/SLED)	In-hospital mortality; In-ICU mortality; 90-day mortality
Zarbock et al. [19]	Single-center, surgical	Germany	231 (112/119)	Yes	67.0	63.2	81.8/19.5/41.6/40.7	32.5	KDIGO Stage 2 AKI (within 8 h) and plasma NGAL > 150 ng/mL	KDIGO stage 3	CRRT	30-/60-/90-day mortality
Gaudry et al. [7]	Multi-center, mixed	France	619 (311/308)	Yes	66.2	65.4	53.0/26.3%/9.0/9.7	79.8	KDIGO Stage 3 AKI (within 6 h)	Severe hyperkalemia (> 6 mmol/L); severe pulmonary edema refractory to diuretics; severe acidosis (pH < 7.15); serum urea > 40 mmol/L; oligo-anuria > 72 h	Mixed (IHD/CRRT)	60-/90-day mortality
Srisawat et al. [20]	Single-center, mixed	Thailand	40 (20/20)	Yes	66.8	55.0	NA	72.5	AKI, any RIFLE stage	Severe metabolic acidosis (pH < 7–20); severe hyperkalemia (> 6 2 mmol/L); severe pulmonary edema refractory to diuretics; persistent oliguria or anuria; serum urea > 40 mg/dL	CRRT	28-day mortality
Lumlertgul et al. [21]	Multicenter, mixed	Thailand	118 (58/60)	Yes	67.1	46.2	44.9/24.6/NA/NA	58.5	Furosemide stress test-nonresponsive patients (UOP < 200 mL in 2 h) (initiation within 6 h); AKI (any stage of KDIGO)	Serum urea > 100 mg/dL; severe hyperkalemia (> 6 mmol/L); severe metabolic acidosis (pH < 715); severe pulmonary edema	CRRT	28-day mortality
Barbar et al. [8]	Multicenter, mixed	France	477 (239/238)	Yes	69.0	60.7	57.8/30.5/8.2/15.6	100.0	Within 12 h after documentation of RIFLE-F	Severe hyperkalemia (> 6 5 mmol/L); severe pulmonary edema refractory to diuretics; severe metabolic acidosis (pH < 715); no renal function recovery after 48 h	Mixed	90-day mortality
Yang et al. [22]	Single-center, mixed	China	142 (71/71)	No	56.7	58.5	37.3/45.8/NA/NA	100	Within 24 h of ICU entry	After 48 h of ICU entry	CRRT	28-day mortality
STARRT-AKI [9]	Multicenter, mixed	Multiple	2927 (1465/1462)	Yes	64.7	68.0	55.9/30.7/13.9/43.9	57.7	Within 12 h of fulfilling eligibility: at least two of the following: twofold increase in serum Cr from baseline; UOP < 6 mL/kg in the preceding 12 h; whole-blood NGAL > 400 ng/mL	Until the development of one or more of the following criteria: a serum potassium level > 6.0 mmol/L, a pH < 7.20 or a serum bicarbonate < 12 mmol/L, severe respiratory failure based on a ratio of the partial pressure of arterial oxygen to the fraction of inspired oxygen of < 200 and clinical perception of volume overload, or persistent AKI for at least 72 h after randomization	Mixed	90-day mortality

AKI, acute kidney injury; CCr, creatinine clearance rate; CKD, chronic kidney disease; Cr, creatinine; CRRT, continuous renal replacement therapy; DM, diabetic mellitus; HTN, hypertension; HF, heart failure; ICU, intensive care unit; IHD. Intermittent hemodialysis; KDIGO, Kidney Disease Improving Global Outcomes; N/A, not available; NGAL, neutrophil gelatinase-associated lipocalin; pre-OP, pre-operation; RIFLE, Risk, Injury, Failure, Loss of kidney function, and End-stage kidney disease; RRT, renal replacement therapy; SLED, Sustained low efficiency dialysis; UOP, urine output; WWS, watchful waiting strategy

*Study design with watchful waiting strategy

### Quality assessment

The Cochrane risk of bias tool was used for quality assessment of RCTs [12]. The following domains were assessed: random sequence generation, allocation concealment, blinding of participants and personnel, blinding of outcome assessment, incomplete outcome data, selective reporting and other bias. The criteria for rating study quality were as follows: high risk study (2 or more items rated as high risk of bias); low risk study (5 or more items rated as low risk and no more than one as high risk); moderate risk study (all remaining situations) [13].

### Definition

Accelerated initiation and standard initiation were defined as relatively earlier versus later hemodialysis according to each study. This study was to investigate the effectiveness of earlier rather than later dialysis. Instead of identifying the point in time of early or late dialysis, we have standardized the terminology in this manuscript to refer to all relatively early dialysis timing as accelerated initiation.

### Outcomes

The primary outcome was all-cause mortality. Secondary outcomes were dialysis dependence and being free of RRT rate after hospital discharge.

### Subgroup analysis

We hypothesized that the following factors could have high impact on patient outcomes observed among different studies: patient population (surgical vs. mixed/medical), disease severity (Sequential Organ Failure Assessment (SOFA) score ≥ 11 vs. < 11), RRT modality (CRRT vs. intermittent hemodialysis [IHD]/mixed), diabetes mellitus prevalence (≥ 35% or < 35%), discrepancy in interval between accelerated and standard initiation time (difference ≥ 24 vs. < 24 h), using comprehensive AKI definitions (with or without including urine amount criteria), single versus multicenter, and sepsis prevalence.

### Data synthesis and statistical analysis

Overall summary log odds ratios (ORs) and 95% CIs were calculated by the Mantel–Haenszel method. The fixed-effects model was used to pool the results of the RCTs. Statistical heterogeneity was assessed by the chi-square test and the *I*^2^ statistic with a *p* < 0.05 or *I*^2^ > 50% was an indication of substantial heterogeneity. In the case of considerable heterogeneity (*I*^2^ > 50% or *p* < 0.05), we performed a sensitivity analysis to detect the influence of a single study on the overall estimate by omitting one study in turn and pooling the remaining ones. In the subgroup analysis, we performed meta-regression to assess the interaction between variables and the timing of RRT initiation on mortality and RRT dependence. Any potential publication bias was assessed by visual assessment of the funnel plots constructed.

We then did the trial sequential analysis (TSA), as well as the sequential monitoring boundaries. The conventional nonsuperiority boundaries were calculated assuming significance levels of 0.05, and a power of 80%. The a-spending boundaries were also calculated using significance levels of 0.01 and 0.05 and the O’Brien-Fleming multiple testing procedure [14]. In order to calculate the neutrality zone, we chose a risk ratio reduction of 20%, because of its compatibility with many trials in the ICU [15], and its representation of an absolute mortality difference of around 10% to 15%, which we considered to be a reasonable effect size. Furthermore, funnel plots were used to evaluate the possibility of publication bias. We used STATA (Version 16, Stata Corp. 2019. College Station, TX: Stata Corp LP) software for the meta-analysis. TSA version 0.9.5.5 (reviewed in November 2016) b software was used for these analyses the cumulative effect of randomized trials on mortality.

## Results

### Search results and study characteristics

The study selection process is summarized in Additional file 1: Figure S1. A total of 25,031 articles were identified through electronic search, and after we excluded duplicate articles and non-relevant articles, the titles and abstracts of the remaining 1071 articles were screened. A total of 56 studies were eligible for full-text reviews, of which 10 RCTs reported data on the timing of RRT initiation; eventually, 4753 critically ill patients with severe AKI were included in our meta-analysis [8, 9, 16–22]. The details of included trials and population characteristics, as well as definitions used for accelerated and standard RRT strategies are shown in Table 1. All 10 studies provided quantifiable results for mortality and RRT dependency during the follow-up period. There were two trials that exclusively enrolled surgical patients, of which one of them were entirely from cardiovascular surgery. The remaining 8 studies enrolled patients from mixed surgical/medical ICU setting. Of the 10 studies, six selected CRRT as the only modality for RRT, and the rest were at the discretion of the attending physicians. Of note, one study applied furosemide stress test before randomizing to either accelerated or standard group, and one study chose to use high level of high AKI biomarker (e.g., NGAL) as criteria for receiving randomization [19, 21].

### Quality of enrolled trials

The quality of enrolled trials varied, and earlier studies tended to lack sufficient information about participants or personnel blinding and concealment process. The studies were published over 18 years and varied in sample sizes (28–2927 patients). TSA was performed on all RCTs using a significance level of 0.05; when the homogeneity of results was considered to be stable, it showed that a total of 8289 patients would be needed to reach a stopping boundary of superiority. However, the Z-curve was parallel to the superior boundary of the accelerated RRT, in term of no superiority to standard RRT and it crossed the neutrality boundary including all trials (Additional file 1: Figure S2).

### Publication bias

The Cochrane Collaboration’s tool for assessing the risk of bias revealed that there were low and/or unclear risk in each study in most domain of bias evaluation (Additional file 1: Figure S3).

The risk of bias was low for random sequence generation in 8 trials (80.0%); allocation concealment in 8 trials (80.0%); blinding of outcome assessment in 10 trials (100.0%); incomplete outcome data in 9 trials (90.0%); selective reporting in 8 trials (80.0%); and other bias (baseline balance) in 7 trials (70.0%). Therefore, according to the criteria of overall quality, 8 trials (80.0%) were rated as low risk studies, 2 trials (20.0%) as moderate, and 0 trial (0.0%) as high (Table 2).

**Table 2:** Summary of included comparative studies for outcome evaluation

References	Population setting and site	Nation, (continence)	HR/OR/RR (CI) for primary endpoint	Urine output (mL/24 h)	AKI definition by stage	Large population (sample ≥ 100)	Time difference between early and late (hour)	Study quality	SOFA score
Bouman et al. [16]	Multicenter mixed	Netherland (Europe)	NA	NA	NA	Small	34.8	High	10.36
Sugahara et al. [17]	Single-center, surgical	Japan (Asia)	NA	NA	NA	Small	2.4	Moderate	NA
Wald et al. [18]	Multicenter, mixed	Canada (North America)	NA	329.8	NA	Large	24	High	12.62
Zarbock et al. [19]	Single-center, surgical	Germany (Europe)	0.66 (0.45–0.97)/–/–	358.7	KDIGO Stage 2	Large	20	High	15.81
Gaudry et al. [7]	Multicenter, mixed	France (Europe)	*1.02 (0.81–1.29)/–/–	NA	KDIGO Stage 3	Large	55	High	10.85
Srisawat et al. [20]	Single-center, mixed	Thailand (Asia)	NA	NA	RIFLE-R	Small	48	High	9.28
Lumlertgul et al. [21]	Multicenter, mixed	Thailand (Asia)	0.96 (0.60–1.53)/–/–	551.1	KDIGO Stage 1	Large	19	High	12.04
Barbar et al. [8]	Multicenter, mixed	France (Europe)	NA	NA	RIFLE-F	Large	45	High	12.3
Yang et al. [22]	Single-center, mixed	China (Asia)	NA	498.9	NA	Large	NA	Moderate	7.35
STARRT-AKI [9]	Multicenter, mixed	Multiple countries (Asia, Europe, North America, Oceania, South America)	–/*1.05 (0.90–1.23)/1.00 (0.93–1.09)	464	KDIGO Stage 2	Large	NA	High	11.7

AKI, acute kidney injury; HR, hazard ratio; KDIGO, Kidney Disease Improving Global Outcome; NA, not available; OR, odds ratio; RIFLE, Risk, Injury, Failure, Loss of kidney function, and End-stage kidney disease RR, relative risk; SOFA, Sequential Organ Failure Assessment

*Adjusted

Funnel plots were used to evaluate the possibility of publication bias. The results showed generally symmetrical distributions for all-cause mortality, dialysis dependence, and free of dialysis (Additional file 1: Figure S4).

### Primary outcomes

**Fig. 1:**
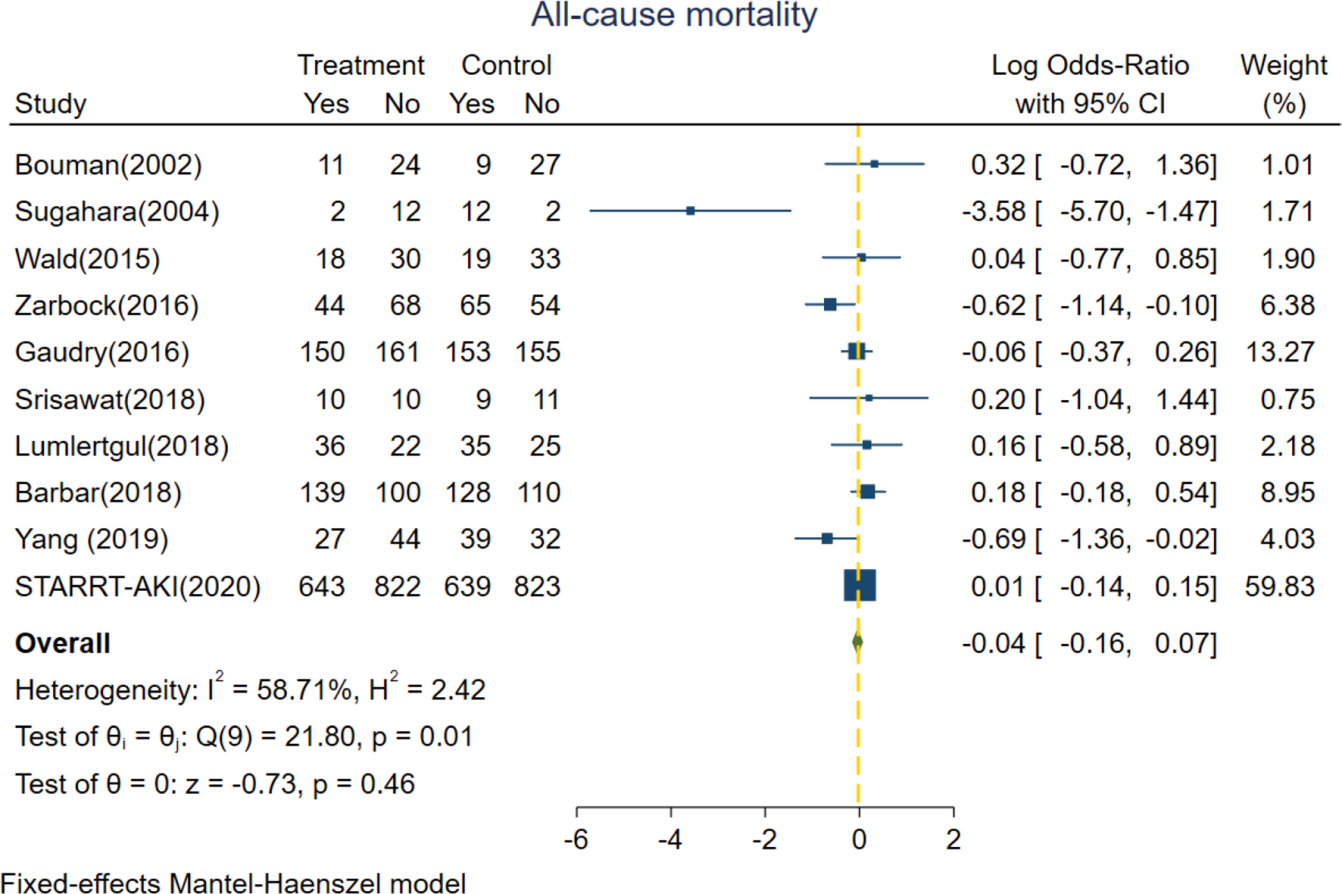
Forest plot for all-cause mortality comparing accelerated versus standard initiation of RRT among RCTs. RCT, randomized controlled trials; RRT, renal replacement therapy

The primary outcome of interest was all-cause mortality, which was based on all trials included and consisted of 4753 patients with 2188 deaths. The pooled mortality rates were 45.5% (1080 of 2373) versus 46.6% (1108 of 2380) in the groups of patients who underwent accelerated versus standard RRT, respectively. No significant survival benefit differences were detected in pooled estimates of included trials between accelerated versus standard RRT group; with a log OR of − 0.04 (95% CI − 0.16 to 0.07, *p* = 0.46) (Fig. 1). High heterogeneity was found among studies. (Fixed effect model, *I*^2^ value of 58.71%; random effect model, *I*^2^ value of 43.49%, Additional file 1: Figure S5) Additionally, there were no significant differences in 28-day and 90-day mortality rates (Additional file 1: Figure S6) between groups with accelerated versus standard initiation of RRT.

### Secondary outcomes

**Fig. 2:**
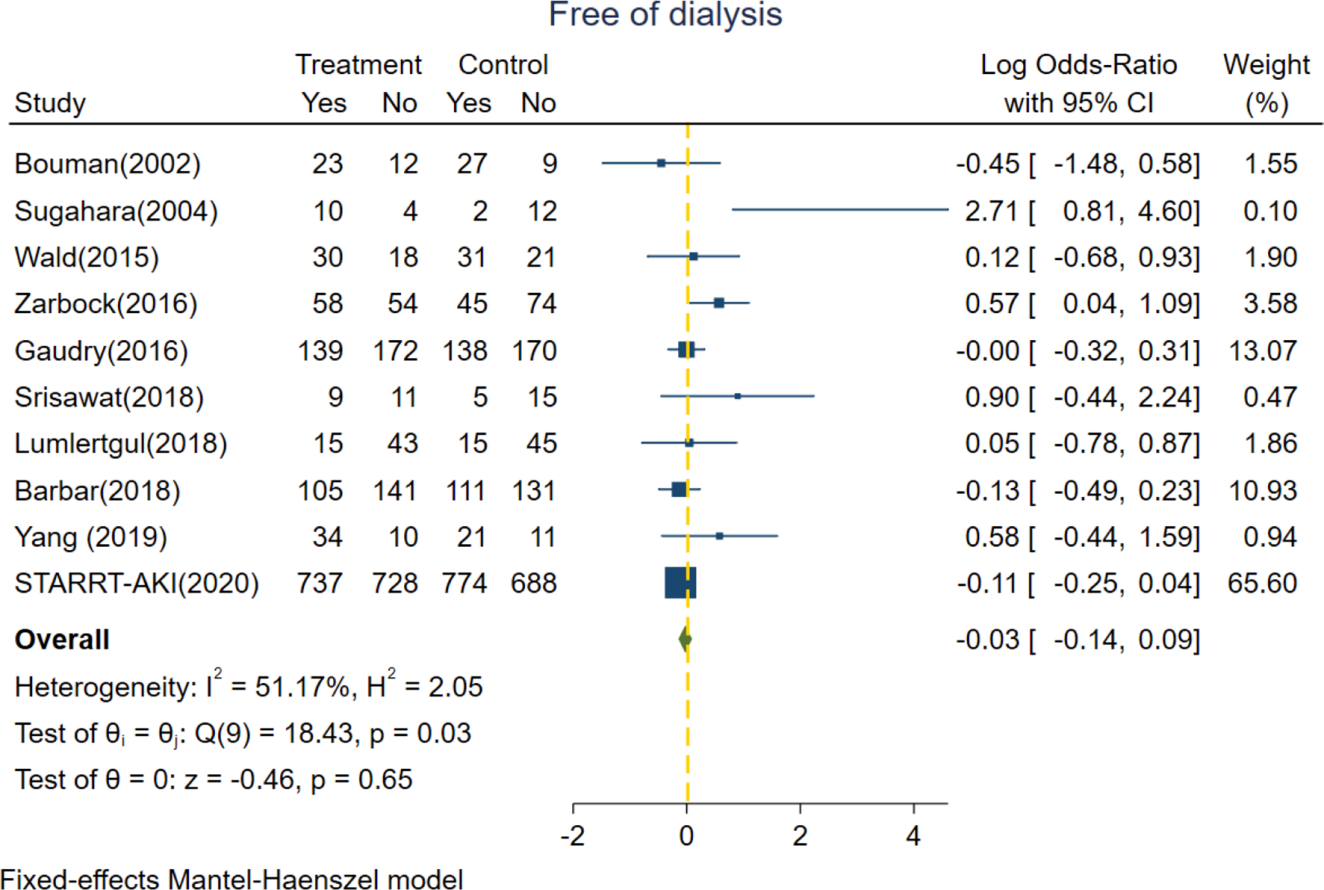
Forest plot for dialysis dependence comparing accelerated versus standard initiation of RRT among RCTs. RCT, randomized controlled trials; RRT, renal replacement therapy

All of the 10 included trials reported the detailed information of free of RRT dependence. In our survey, there were 48.9% (1160/2380) and 49.1% (1169/2384) of severe AKI patients randomized into the accelerated group and standard group, respectively, who did not end up receiving RRT. Among the survivors post discharge, there were 89.7% (1160/1293) and 91.9% (1169/1272) of patients who showed eventual spontaneous renal recovery and did not need long-term RRT in the accelerated group and standard group, respectively. The pooled rates of being free of dialysis showed no statistical significance between accelerated and standard groups with a log OR of − 0.03 (95% CI − 0.14 to 0.09, *p* = 0.65) under fixed effect model (*I*^2^ value = 51.17%) (Fig. 2).

**Fig. 3:**
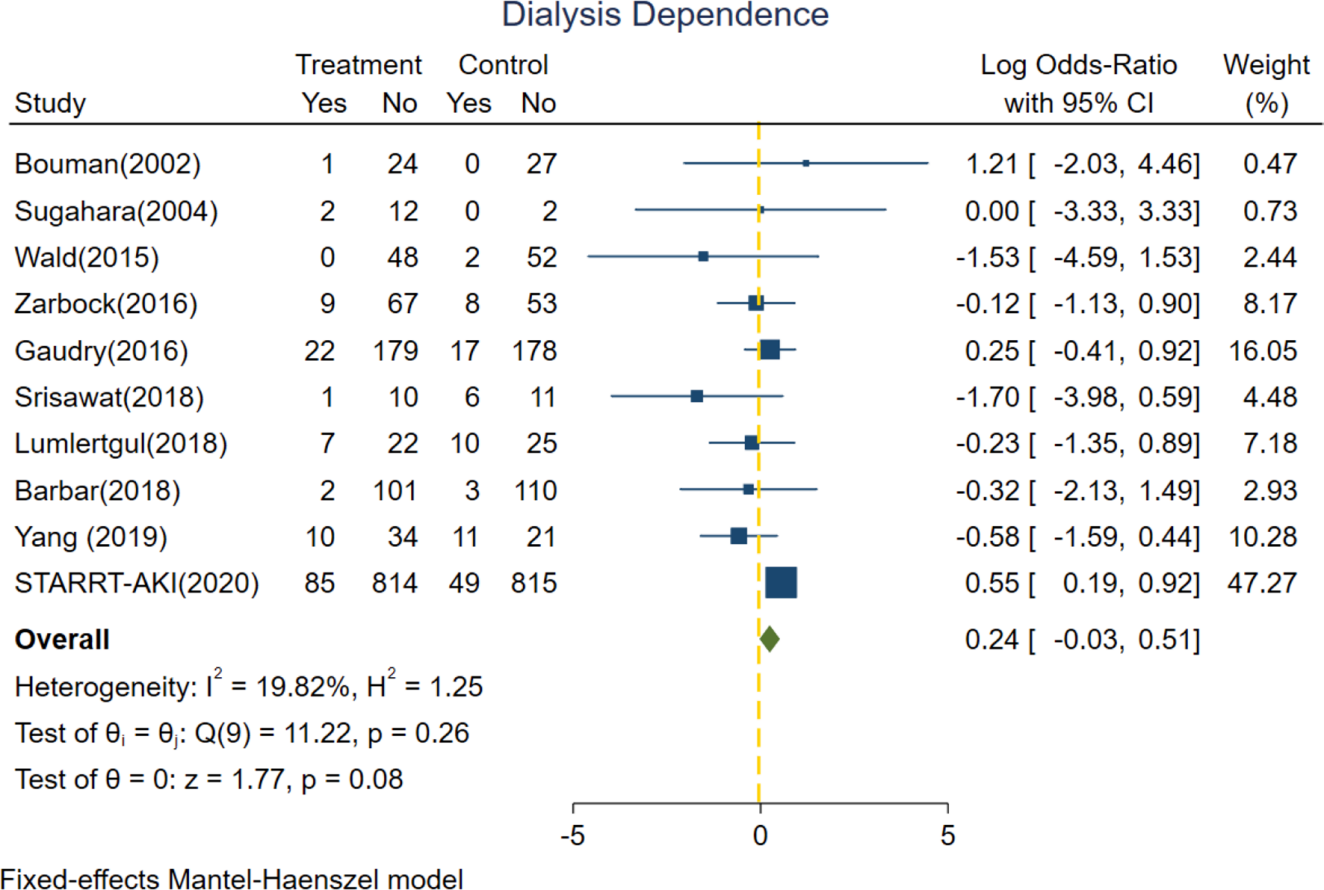
Forest plot for free of dialysis comparing accelerated versus standard initiation of RRT among RCTs. RCT, randomized controlled trials; RRT, renal replacement therapy

The 10 trials included also reported the detailed information of dialysis dependence. Figure 3 shows no significant difference between risk of RRT dependence between accelerated group and standard group (log OR = 0.24, 95% CI − 0.03 to 0.51, *p* = 0.08) under fixed effect model with low heterogeneity (*I*^2^ value = 19.82%).

### Subgroup analysis

**Fig. 4:**
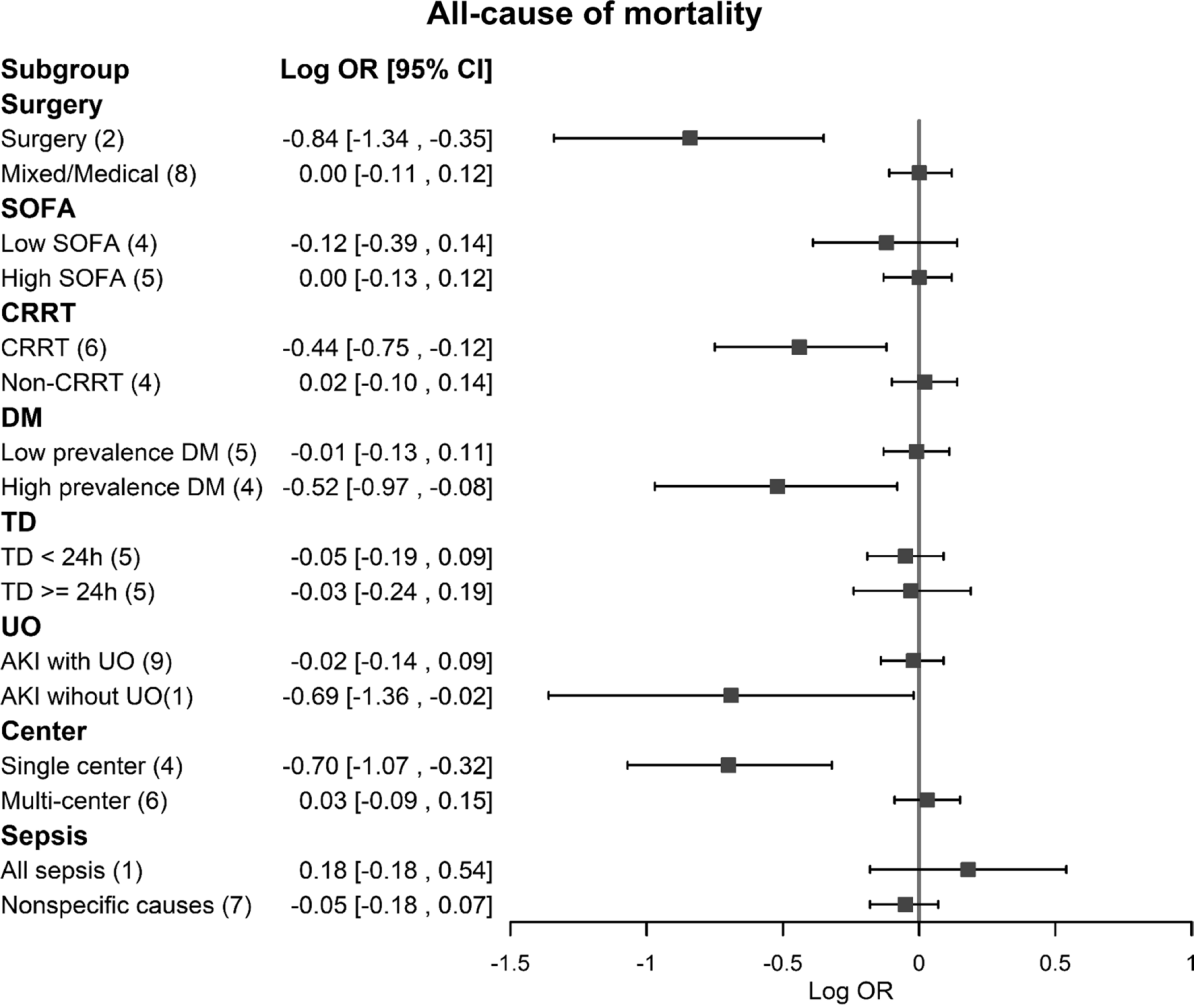
Forest plots of subgroups for all-cause mortality comparing accelerated versus standard initiation of RRT among RCTs. RCT, randomized controlled trials; RRT, renal replacement therapy

**Fig. 5:**
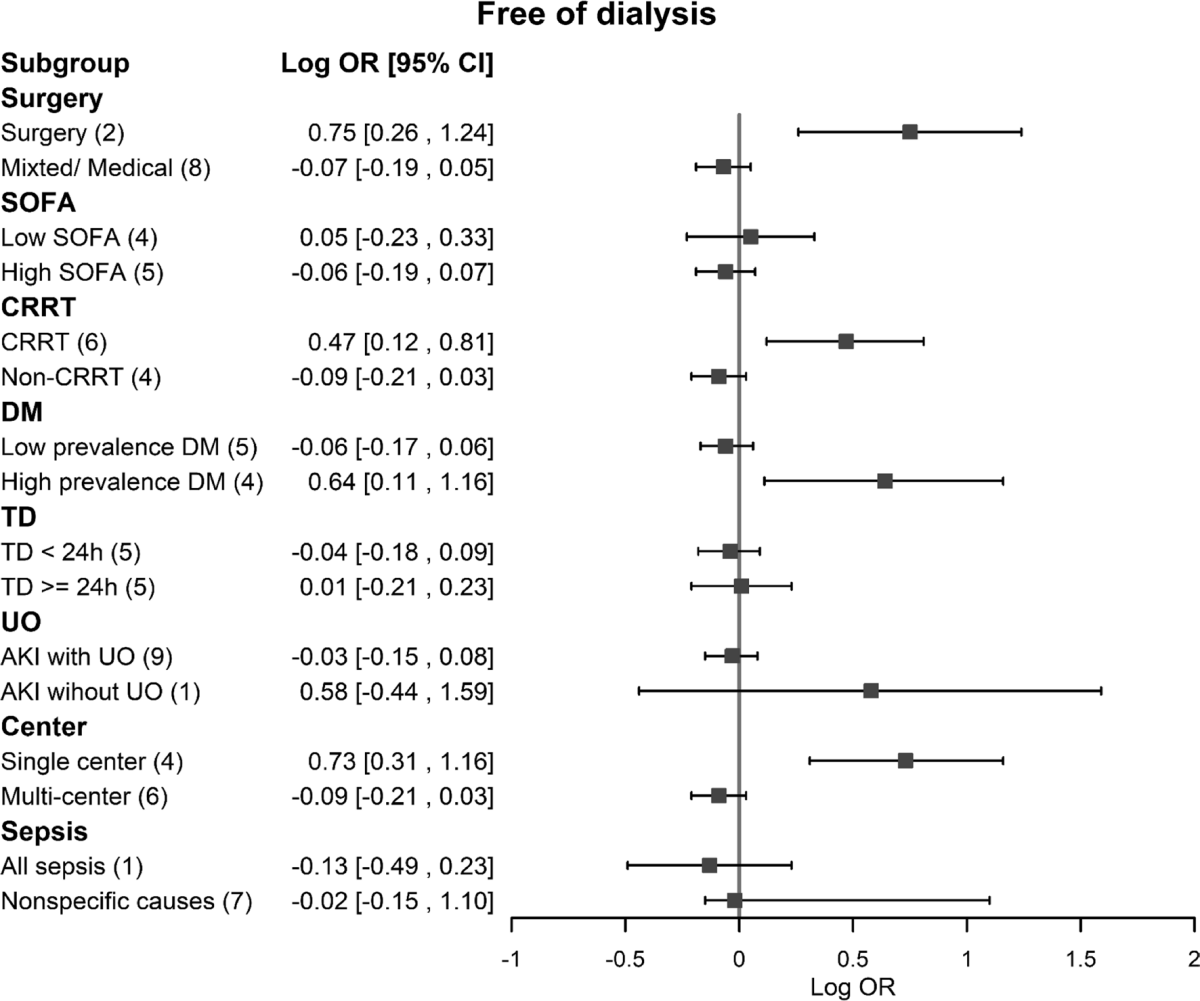
Forest plots of subgroups for free of dialysis comparing accelerated versus standard initiation of RRT among RCTs. RCT, randomized controlled trials; RRT, renal replacement therapy

In our subgroup analyses, the association between accelerated initiation of RRT and a lower risk of all-cause mortality was significant in the setting of surgical ICU patients, CRRT modality and single center settings (Fig. 4). On the other hand, the association between accelerated initiation of RRT and a higher risk of dialysis dependence was also significant in the setting of high disease severity with SOFA score ≥ 11, mixed RRT modality, lower DM prevalence, interval time between accelerated and standard initiation being less than 24 h, using AKI definitions including urine output and multicenter studies (Additional file 1: Figure S7). We further investigated the possible effect modification of the potential variables for free from dialysis. It was shown that surgical ICU patients, using CRRT modality, high DM prevalence and single center settings also played positive associations between accelerated initiation of RRT strategy and higher chance of being free of dialysis (Fig. 5).

### Sensitivity analysis

Different models were used to evaluate the primary and secondary outcomes. There were no significant differences in all cause-mortality, dialysis dependence and free of dialysis between accelerated and standard groups in the pooled data of the 10 studies in random effects model (Additional file 1: Figures S5, S8, S9). In five RCTs that provided the adjusted hazard ratio (HR) in regard to patient mortality, the risk of all-cause mortality in accelerated RRT was similar to that of standard RRT (adjusted HR = 1.02, 95% CI 0.94–1.09, *p* < 0.01, Additional file 1: Figure S10).

Among the 10 RCTs, the definition of accelerated/early and standard/delayed initiation of RRT of the study by Gaudry was quite different from that of other RCTs, and the study by Yang was not a SCI (science citation index) article. After excluding the two studies, the pooled data of the remaining 8 RCTs revealed similar results in primary and secondary outcomes (Additional file 1: Figures S11–S13). Consistent with our main results, the critically ill AKI patients could benefit from accelerated RRT regarding all-cause mortality and free of dialysis only if they were surgical ICU patients or underwent CRRT treatment. The findings were not materially different from the standard analysis and remained robust in the sensitivity analyses. Additionally, the sensitivity analysis revealed that the results were not altered when removing anyone study (data not shown).

### Assessment of evidence quality and summary of findings.

Evidence quality assessment was performed using the GRADE system (Additional file 1: Appendix 7). We evaluated the primary outcome and secondary outcomes and presented as a summary of findings in supplementary file.

## Discussion

In a systematic review of 10 RCTs including 4753 critically ill patients with severe AKI, we did not find significant survival benefits (28-day nor 90-day mortality) in patients who underwent accelerated versus standard RRT. For those critical AKI patients who underwent CRRT treatment or were in the surgical ICU setting, accelerated RRT showed survival benefits as well as more free of dialysis. However, the relative risk of dialysis dependence increased in accelerated RRT group, when those AKI patients were non-CRRT and of high disease severity groups. To our knowledge, this is the most comprehensive systematic review to date that included the highest number of RCTs and the largest number of critically ill AKI patients.

Even though the literatures addressing this comparison were highly heterogeneous, our funnel meta-regression analysis showed only limited publication bias. Our TSA showed a constant result and low risk of bias among these randomized studies comparing the impact on mortality between accelerated versus standard initiation of dialysis in critically ill AKI patients. Furthermore, the total number of patients is enough to achieve a confident conclusion because the Z-curve did cross the neutrality line from the TSA.

### Being free of dialysis

Contrary to the previous reports, we did not find a significant effect of standard dialysis leading to a higher rate of being free of RRT when all patient populations were considered; however, in critically ill AKI patients with higher disease severity or who underwent CRRT treatment, there were higher rates of being free of dialysis among the survivors.

Some large observational studies, that only included patients who were receiving RRT, suggest that CRRT is an independent predictor of renal recovery among survivors [23, 24]. CRRT could permit slow but continuous removal of solutes and water, thereby conferring better hemodynamic tolerability. Relative hemodynamic stability during CRRT sessions, compared to intermittent dialysis, could mitigate occult kidney injury. We showed the evidence to elucidate the impact of choice of therapy on this outcome from RCTs [25].

### Dialysis dependence

Another conclusion to result from this meta-analysis is that about 90.8% (89.7% in accelerated and 91.9% in standard group) of the survivors with severe AKI in our survey did not undergo dialysis at the end of the studies, due to the fortunate spontaneous recovery of renal function from managing underlying etiologies and/or accompanying co-morbidities which occurred within hours to days.

To our knowledge, our study is the largest systematic review to date to address the question of optimal timing regarding RRT initiation and its impact on patient survival and dialysis dependence that included several recent elegant large RCTs [7–9, 19]. Furthermore, our finding is consistent in 73.3% of RCTs when watchful waiting strategy was adopted into the study design. For the sake of all-cause mortality, it is startling to find 90.8% of the survivors to avoid the potential risks of an extracorporeal support technique if they do not actually need it, not to mention the savings to be yielded in terms of medical cost, time, man-power and capacity of the ICU.

A delayed strategy for the initiation of RRT could allow time for stabilization of the patients’ condition, thus enabling starting of RRT when the patients were more hemodynamically stable, or even precluding the need for such therapy if the renal function recovers spontaneously that contributed to free of dialysis. The results of the BICAR-ICU study [26] suggested that optimizing medical treatment can avoid RRT for severely metabolically acidotic AKI cases, and thus reducing mortality. Moreover, the time needed for most inotropes to reach their efficacy is less than 28.1 h, which is the average difference in timing between these accelerated versus standard initiation. This implicated that the patients who would have to utilize RRTs were at a more hemodynamically stable state. Although we still lack miracle drug therapies capable of blocking or reversing severe AKI, accelerated RRT might not be the solution to all critical AKI patients.

### Subgroup analyses

Previous investigation had looked at special populations such as sepsis and found no significant difference in survival of AKI patients according to strategy for the initiation of RRT [27]. In the current study, we further tried to explore the clinical impact of some other potential factors, such as different study settings, disease severities, diabetic percentage, and dialysis discrepancy time less than 24 h. In our subgroup analyses, we found no survival differences between accelerated versus standard RRT initiation time even after multivariate adjustment. We also found that the values of combining serum creatinine and urine output (which are components of the modern RIFLE and KDIGO criteria), as well as percentages of the septic patients had no significant impact on patient outcomes, and therefore they should not be used to decide on the time of RRT initiation.

There are some possible explanations for the discordance and heterogeneity among different studies. Using varying AKI definitions and different AKI stage criteria for RRT initiation could account for part of the observed heterogeneities. For most of the previously reported cohort studies, the differences in pre-intervention study groups contributed to the heterogeneity of the results, and therefore made the systematic reviews difficult to interpret.

### Strengths and limitations

The strength of our present analysis rests on our extensive literature search on related RCTs. We used standard Cochrane protocols and had the largest cumulative RCTs study sample size to date in comparison with the previous reports. We only focused on the RCTs that had a reasonable quality with limited differential dropout based on the assigned treatment arm. One of the differences that our study has in comparison with previous reports is the inclusion of the recently published elegant RCT studies with large patient numbers and global multi-national inclusion in patients’ recruiting [9], especially its effect on free of dialysis. These recently published RCTs that included watchful waiting strategies were not included in prior meta-analysis; this accounts for the differences in our results from those of earlier authors [28–31]. The strength of our meta-analysis also lies in comprehensive data search with subgroup analyses across several clinical scenarios. We adapted the GRADE approach to rate the certainty of evidence [32].

Protocolizing the optimal timing of RRT for all AKI patients may be too crude and imprecise, in the era of modern personalized medicine. It could be more important to give clinicians reliable information about when to initialize RRT in certain precisely defined patient groups. The negative result on the primary endpoint turns out to be hiding among a high level of heterogeneity in terms of disease progression, that could not be accurately predicted by the staging of AKI at the time of inclusion.

In our systematic review, we found no further information regarding the other factors associated with mortality, and therefore, we cannot comment on differences in the outcomes on the basis of one single intervention, i.e., accelerated or standard dialysis initiation. There were only two trials that exclusively enrolled surgical patients, of which one of them were entirely from cardiovascular surgery. Furthermore, no trial standardized the dialysis modality or dose delivered during initializing RRT. We were not able to access the unpublished reports, e.g., negative results of accelerated RRT which might have biased our results. Although our funnel meta-regression and Cochrane Collaboration’s tool analysis showed a limited publication bias (supplementary figures), the bias is always difficult to ascertain with a small sample size of the included studies. Finally, the definition of “accelerated” RRT was variable and may have unduly influenced pooled effect estimates. The timing of RRT defined by traditional markers was relatively late which may influence the effectiveness of the early treatment. In our TSA, we included trials of patients without severe AKI, which yielded enough information size to conclude that accelerated RRT probably does not benefit patients (due to the *Z* curve crossing the neutrality line). Nonetheless, our conclusion yielded from studies that consisted of different study designs and different clinical scenarios. Of note, we just raised the possibility of the timing of RRT defined by traditional markers was relatively late, which may influence the effectiveness of the early treatment. Our intention was to investigate whether receiving RRT earlier than the traditional timing would influence its effectiveness. Further research efforts are certainly needed for the pursuit of better precision medicine. It could be more fruitful to investigate if different etiologies of AKI (pre-renal vs. renal vs. obstructive, cardiogenic shock, hypovolemic shock, sepsis-related, etc.) affect outcomes of accelerated versus standard RRT; and to evaluate if the efficacy of CRRT fits into various underlying causes of AKI in critically ill patients. These issues can be incorporated into the design of future RCTs in evaluating the optimal timing and modalities of RRT for critically ill AKI patients, in order to reach a new horizon and higher success rate of treatment in this field. Moreover, further investigations into improvement in treatments to resuscitate the patients’ hemodynamic stability and to manage each underlying mechanisms of AKI might contribute to mitigate the current extremely high mortality rate of these critically ill patients with RRT-requiring AKI.

### Conclusion

Accelerated dialysis initiation in critically ill patients with severe AKI does not decrease mortality, alter the possibility from free of dialysis, or mitigate dialysis dependence among survivors as compared with the standard RRT initiation strategy. However, in patients who underwent CRRT treatment or were in the surgical ICU setting, accelerated RRT could benefit the possibility of survival and free of dialysis. However, accelerated RRT initiation could be associated with higher risk of dialysis dependence when those severe AKI patients were treated with non-CRRT modality or were of high disease severity.

## Supplementary information

**Additional file 1:** Supplementary appendix.

## Data Availability

The datasets used and/or analyzed during the current study are available from the corresponding author on reasonable request.

## Abbreviations

AKI: Acute kidney injury
CI: Confidence intervals
CRRT: Continuous renal replacement therapy
DM: Diabetes mellitus
FST: Furosemide stress test (FST)
ICU: Intensive care units
IHD: Intermittent hemodialysis (IHD)
OR: Odds-ratio
PRISMA: Preferred Reporting Items of Systematic Reviews and Meta-Analyses
RCTs: Randomized controlled trials
RRT: Renal replacement therapy
TSA: Trial sequential analysis
WWS: Watchful waiting strategy
