# Supplementary material for: Accelerated versus standard initiation of renal replacement therapy for critically ill patients with acute kidney injury: a systematic review and meta-analysis of RCT studies
Source: Crit Care. 2021 Jan 5;25:5. doi: 10.1186/s13054-020-03434-z (PMC7784335; doi:10.1186/s13054-020-03434-z)
Supplement: Supplementary file 1 — Additional file 1: Supplementary appendix. [file 13054_2020_3434_MOESM1_ESM.pdf]

Supplementary appendix

This supplementary appendix provides:

1. Search equation via PubMed, EMBASE, MEDLINE, and Cochrane library
2. Quality assessment of the included studies.
3. PRISMA checklist.
4. Subgroup analysis of the forest plot of odds ratio (OR) and 95% confidence interval (CI).
5. Summary of contextual factor data.
6. PROSPERO protocol registration.
7. Quality assessment the GRADE results.
8. Flow chart showing Search strategy for studies in China.

1. Search equation via PubMed, EMBASE, MEDLINE, and Cochrane library

Appendix.

Search strategies for the different databases ran on July 20, 2020.

PubMed

#1 ((((((((((("acute kidney injury"[MeSH Terms] OR (("acute"[All Fields] AND "kidney"[All Fields]) AND "injury"[All Fields])) OR "acute kidney injury"[All Fields]) OR (((("acute kidney injury"[MeSH Terms] OR (("acute"[All Fields] AND "kidney"[All Fields]) AND "injury"[All Fields])) OR "acute kidney injury"[All Fields]) OR (("acute"[All Fields] AND "renal"[All Fields]) AND "failure"[All Fields])) OR "acute renal failure"[All Fields])) OR (("acute kidney injury"[MeSH Terms] OR (("acute"[All Fields] AND "kidney"[All Fields]) AND "injury"[All Fields])) OR "acute kidney injury"[All Fields])) OR (((("acute kidney injury"[MeSH Terms] OR (("acute"[All Fields] AND "kidney"[All Fields]) AND "injury"[All Fields])) OR "acute kidney injury"[All Fields]) OR (("acute"[All Fields] AND "kidney"[All Fields]) AND "failure"[All Fields])) OR "acute kidney failure"[All Fields])) OR (("anuria"[MeSH Terms] OR "anuria"[All Fields]) OR "anurias"[All Fields])) OR ("oliguria"[MeSH Terms] OR "oliguria"[All Fields])) OR (("acidosis"[MeSH Terms] OR "acidosis"[All Fields]) OR "acidoses"[All Fields])) AND "hasabstract"[All Fields]) AND ((((((((((("timely"[All Fields] OR "timing"[All Fields]) OR "timings"[All Fields]) OR ("time"[MeSH Terms] OR "time"[All Fields])) OR ((("start"[All Fields] OR "started"[All Fields]) OR "starting"[All Fields]) OR "starts"[All Fields])) OR (((((((((("initial"[All Fields] OR "initially"[All Fields]) OR "initials"[All Fields]) OR "initiate"[All Fields]) OR "initiated"[All Fields]) OR "initiates"[All Fields]) OR "initiating"[All Fields]) OR "initiation"[All Fields]) OR "initiations"[All Fields]) OR "initiator"[All Fields]) OR "initiators"[All Fields])) OR (((("begin"[All Fields] OR "begining"[All Fields]) OR "beginning"[All Fields]) OR "beginnings"[All Fields]) OR "begins"[All Fields])) OR "early"[All Fields]) OR "late"[All Fields]) OR (((("begin"[All Fields] OR "begining"[All Fields]) OR "beginning"[All Fields]) OR "beginnings"[All Fields]) OR "begins"[All Fields])) OR ((((("commence"[All Fields] OR "commenced"[All Fields]) OR "commencement"[All Fields]) OR

"commencements"[All Fields]) OR "commences"[All Fields]) OR "commencing"[All Fields])) OR ((((((((("accelerate"[All Fields] OR "accelerated"[All Fields]) OR "accelerates"[All Fields]) OR "accelerating"[All Fields]) OR "acceleration"[MeSH Terms]) OR "acceleration"[All Fields]) OR "accelerations"[All Fields]) OR "accelerator"[All Fields]) OR "accelerator s"[All Fields]) OR "accelerators"[All Fields])) AND "hasabstract"[All Fields])) AND (((((((((((((((((((((((((((((((((((((("dialysance"[All Fields] OR "dialysances"[All Fields]) OR "dialysation"[All Fields]) OR "dialysator"[All Fields]) OR "dialysators"[All Fields]) OR "dialyse"[All Fields]) OR "dialysed"[All Fields]) OR "dialyser"[All Fields]) OR "dialysers"[All Fields]) OR "dialysing"[All Fields]) OR "dialysis solutions"[Pharmacological Action]) OR "dialysis solutions"[MeSH Terms]) OR ("dialysis"[All Fields] AND "solutions"[All Fields])) OR "dialysis solutions"[All Fields]) OR "dialysate"[All Fields]) OR "dialysates"[All Fields]) OR "dialyzates"[All Fields]) OR "dialysis"[MeSH Terms]) OR "dialysis"[All Fields]) OR "dialyses"[All Fields]) OR "dialyzability"[All Fields]) OR "dialyzable"[All Fields]) OR "dialyzate"[All Fields]) OR "dialyzation"[All Fields]) OR "dialyze"[All Fields]) OR "dialyzed"[All Fields]) OR "dialyzer"[All Fields]) OR "dialyzer s"[All Fields]) OR "dialyzers"[All Fields]) OR "dialyzing"[All Fields]) OR "renal dialysis"[MeSH Terms]) OR ("renal"[All Fields] AND "dialysis"[All Fields])) OR "renal dialysis"[All Fields]) OR (("renal replacement therapy"[MeSH Terms] OR (("renal"[All Fields] AND "replacement"[All Fields]) AND "therapy"[All Fields])) OR "renal replacement therapy"[All Fields])) OR (((("haemodialysis"[All Fields] OR "renal dialysis"[MeSH Terms]) OR ("renal"[All Fields] AND "dialysis"[All Fields])) OR "renal dialysis"[All Fields]) OR "hemodialysis"[All Fields])) OR (((("haemofiltration"[All Fields] OR "hemofiltration"[MeSH Terms]) OR "hemofiltration"[All Fields]) OR "hemofiltrated"[All Fields]) OR "hemofiltrations"[All Fields])) OR (("haemodiafiltration"[All Fields] OR "hemodiafiltration"[MeSH Terms]) OR "hemodiafiltration"[All Fields])) AND (((((((("acute kidney injury"[MeSH Terms] OR (("acute"[All Fields] AND "kidney"[All Fields]) AND "injury"[All Fields])) OR "acute kidney injury"[All Fields]) OR (((("acute kidney injury"[MeSH Terms] OR (("acute"[All Fields] AND "kidney"[All Fields]) AND "injury"[All Fields])) OR "acute kidney injury"[All Fields]) OR (("acute"[All Fields] AND "renal"[All Fields]) AND "failure"[All Fields])) OR "acute renal failure"[All Fields])) OR (("acute kidney injury"[MeSH Terms] OR (("acute"[All Fields] AND "kidney"[All Fields]) AND

"injury"[All Fields])) OR "acute kidney injury"[All Fields])) OR (((("acute kidney injury"[MeSH Terms] OR (("acute"[All Fields] AND "kidney"[All Fields]) AND "injury"[All Fields])) OR "acute kidney injury"[All Fields]) OR (("acute"[All Fields] AND "kidney"[All Fields]) AND "failure"[All Fields])) OR "acute kidney failure"[All Fields])) OR (("anuria"[MeSH Terms] OR "anuria"[All Fields]) OR "anurias"[All Fields])) OR ("oliguria"[MeSH Terms] OR "oliguria"[All Fields])) OR (("acidosis"[MeSH Terms] OR "acidosis"[All Fields]) OR "acidoses"[All Fields]))) AND (((((((((("timely"[All Fields] OR "timing"[All Fields]) OR "timings"[All Fields]) OR ("time"[MeSH Terms] OR "time"[All Fields])) OR ((("start"[All Fields] OR "started"[All Fields]) OR "starting"[All Fields]) OR "starts"[All Fields])) OR ((((((((("initial"[All Fields] OR "initially"[All Fields]) OR "initials"[All Fields]) OR "initiate"[All Fields]) OR "initiated"[All Fields]) OR "initiates"[All Fields]) OR "initiating"[All Fields]) OR "initiation"[All Fields]) OR "initiations"[All Fields]) OR "initiator"[All Fields]) OR "initiators"[All Fields])) OR (((("begin"[All Fields] OR "begining"[All Fields]) OR "beginning"[All Fields]) OR "beginnings"[All Fields]) OR "begins"[All Fields])) OR "early"[All Fields]) OR "late"[All Fields]) OR (((("begin"[All Fields] OR "begining"[All Fields]) OR "beginning"[All Fields]) OR "beginnings"[All Fields]) OR "begins"[All Fields])) OR ((((("commence"[All Fields] OR "commenced"[All Fields]) OR "commencement"[All Fields]) OR "commencements"[All Fields]) OR "commences"[All Fields]) OR "commencing"[All Fields])) OR ((((((((("accelerate"[All Fields] OR "accelerated"[All Fields]) OR "accelerates"[All Fields]) OR "accelerating"[All Fields]) OR "acceleration"[MeSH Terms]) OR "acceleration"[All Fields]) OR "accelerations"[All Fields]) OR "accelerator"[All Fields]) OR "accelerator s"[All Fields]) OR "accelerators"[All Fields]))) AND "hasabstract"[All Fields]))

EMBASE

#1 'acute kidney failure'/de AND ('renal replacement therapy'/de OR 'hemodialysis'/de OR 'hemofiltration'/de OR 'hemodiafiltration'/de OR 'dialysis'/exp OR 'dialysis'/de) AND ('timing'/exp OR timing OR time OR begin OR beginning OR commencement OR start OR early OR late OR optimal OR accelerated OR standard OR initiation OR initiated OR delayed)

Cochrane library

#1 MeSH descriptor: [Acute Kidney Injury] explode all trees

#2 MeSH descriptor: [Acute Kidney Injury] explode all trees

#3 MeSH descriptor: [Acidosis] explode all trees

#4 MeSH descriptor: [Oliguria] explode all trees

#5 #1 or #2 or #3 or #4

#6 MeSH descriptor: [Renal Replacement Therapy] explode all trees

#7 MeSH descriptor: [Renal Dialysis] explode all trees

#8 MeSH descriptor: [Dialysis] explode all trees

#9 MeSH descriptor: [Hemofiltration] explode all trees

#10 MeSH descriptor: [Hemodiafiltration] explode all trees

#11 #6 or #7 or #8 or #9 or #10

#12 timing or time or beginning or start or commencement or initiate or initiated or standard or early or late or optimal

#13 #5 and #11 and #12

2. Quality assessment of the included studies

Supplemental Table 1. Newcastle-Ottawa Scale Quality Assessment of included studies

	Selection				Comparability		Outcomes			
**First author / Year**	**Representativeness of the exposed cohort**	**Selection of the non exposed cohort**	**Ascertainment of exposure to implants**	**Demonstration that outcome of interest was not present at the start of study**	**Comparability: Age and Gender**	**Comparability: Additional Factors**	**Assessment of outcome**	**Was follow up long enough for outcomes to occur**	**Adequacy of follow up of cohorts**	**Total**
**Bouman/2002**	★	★	★		★	★	★	★	★	8
**Sugahara/2004**	★	★	★		★	★	★	★	★	8
**Wald/2015**	★	★	★		★	★	★	★	★	8
**Zarbock/2016**	★	★	★		★	★	★	★	★	8
**Gaudry/2016**	★	★	★		★	★	★	★	★	8
**Lemlertgul/2018**	★	★	★		★	★	★		★	7
**Srisawat/2018**	★	★	★			★	★		★	6
**Barber/2018**	★	★	★		★	★	★	★	★	8
**Yang/2019**	★	★	★		★	★	★		★	7
**STARRT-AKI/2020**	★	★	★		★	★	★	★	★	8

3. PRISMA checklist

Section/topic	#	Checklist item	Reported on page #
**TITLE**			
Title	1	Identify the report as a systematic review, meta-analysis, or both.	1
**ABSTRACT**			
Structured summary	2	Provide a structured summary including, as applicable: background; objectives; data sources; study eligibility criteria, participants, and interventions; study appraisal and synthesis methods; results; limitations; conclusions a implications of key findings; systematic review registration number.	4-5
**INTRODUCTION**			
Rationale	3	Describe the rationale for the review in the context of what is already known.	7
Objectives	4	Provide an explicit statement of questions being addressed with reference to participants, interventions, comparisons, outcomes, and study design (PICOS).	8-9
**METHODS**			
Protocol and registration	5	Indicate if a review protocol exists, if and where it can be accessed (e.g., Web address), and, if available, prov e	10-11
Eligibility criteria	6	Specify study characteristics (e.g., PICOS, length of follow-up) and report characteristics (e.g., years considered, language, publication status) used as criteria for eligibility, giving rationale.	10
Information sources	7	Describe all information sources (e.g., databases with dates of coverage, contact with study authors to identify additional studies) in the search and date last searched.	10
Search	8	Present full electronic search strategy for at least one database, including any limits used, such that it could be repeated.	10, Appendix
Study selection	9	State the process for selecting studies (i.e., screening, eligibility, included in systematic review, and, if applicable included in the meta-analysis).	11
Data collection process	10	Describe method of data extraction from reports (e.g., piloted forms, independently, in duplicate) and any proces es	12
Data items	11	List and define all variables for which data were sought (e.g., PICOS, funding sources) and any assumptions ar simplifications made.	12
Risk of bias in individual studies	12	Describe methods used for assessing risk of bias of individual studies (including specification of whether this wa done at the study or outcome level), and how this information is to be used in any data synthesis.	12-13
Summary measures	13	State the principal summary measures (e.g., risk ratio, difference in means).	13
Synthesis of results	14	Describe the methods of handling data and combining results of studies, if done, including measures of consiste cy	13-14

Section/topic	#	Checklist item	Reported on page #
Risk of bias across studies	15	Specify any assessment of risk of bias that may affect the cumulative evidence (e.g., publication bias, selective reporting within studies).	14
Additional analyses	16	Describe methods of additional analyses (e.g., sensitivity or subgroup analyses, meta-regression), if done, indicating	13-14
**RESULTS**			
Study selection	17	Give numbers of studies screened, assessed for eligibility, and included in the review, with reasons for exclusions at	14, Fig 1
Study characteristics	18	For each study, present characteristics for which data were extracted (e.g., study size, PICOS, follow-up period) and provide the citations.	14-15, Table 1-2
Risk of bias within studies	19	Present data on risk of bias of each study and, if available, any outcome level assessment (see item 12).	18, Appendix
Results of individual studies	20	For all outcomes considered (benefits or harms), present, for each study: (a) simple summary data for each intervention group (b) effect estimates and confidence intervals, ideally with a forest plot.	15-18, Table 2 S-Fig 1,
Synthesis of results	21	Present results of each meta-analysis done, including confidence intervals and measures of consistency.	16-18, Table 3 Figs 2-3,5 S-Fig 4.5
Risk of bias across studies	22	Present results of any assessment of risk of bias across studies (see Item 15).	18, S-Fig 2,3
Additional analysis	23	Give results of additional analyses, if done (e.g., sensitivity or subgroup analyses, meta-regression [see Item 16]).	16-17, Figs 4
**DISCUSSION**			
Summary of evidence	24	Summarize the main findings including the strength of evidence for each main outcome; consider their relevance o	18-19
Limitations	25	Discuss limitations at study and outcome level (e.g., risk of bias), and at review-level (e.g., incomplete retrieval c identified research, reporting bias).	24-25
Conclusions	26	Provide a general interpretation of the results in the context of other evidence, and implications for future researc	25-26
**FUNDING**			

Funding	27	Describe sources of funding for the systematic review and other support (e.g., supply of data); role of funders for the systematic review.	27

4. Supplemental Figures

Supplemental Figure 1. Flowchart of study selection for meta-analysis

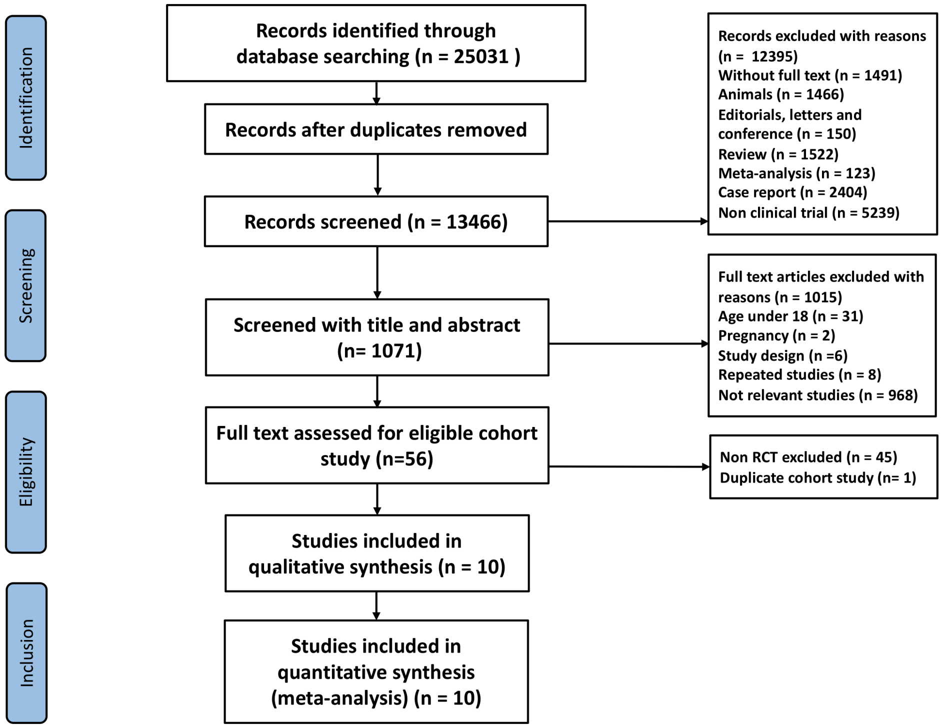

Abbreviations: RCT: randomized controlled trials.

已删除: .

已删除:

<物件>

已删除:

已删除:

Supplemental Figure 2. Trial sequential analysis of low risk of bias randomized studies comparing the impact on mortality of accelerated versus standard initiation of renal replacement therapy (RRT) for critically-ill patients with acute kidney injury. The boundaries for benefit are not crossed and no effect on mortality is observed; the Z-curve is parallel to the boundary of the accelerated RRT.

已刪除: 1

已刪除:

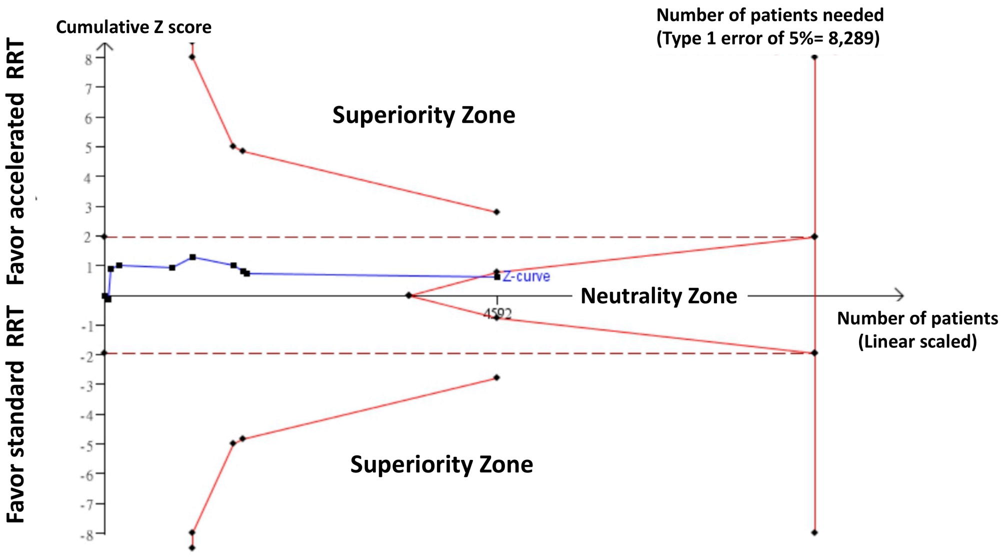

Supplemental Figure 3. Risk of bias in individual studies. The risk of allocation concealment, the risk of blinding of participants and personnel, and the risk of blinding of outcome assessment were classified as high or unclear.

(A)

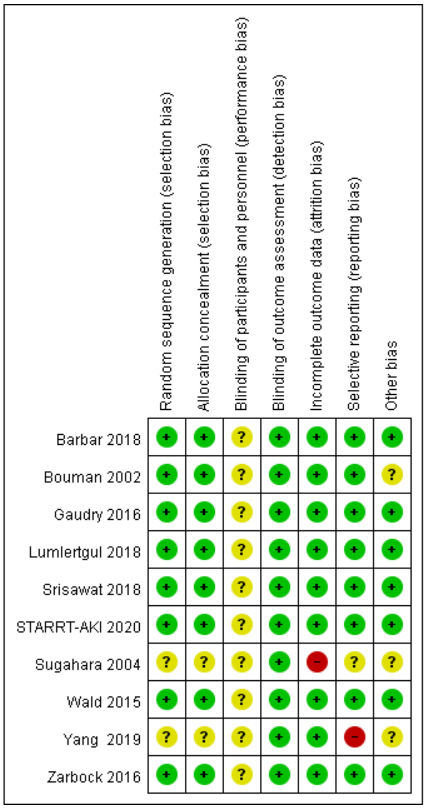

(B)

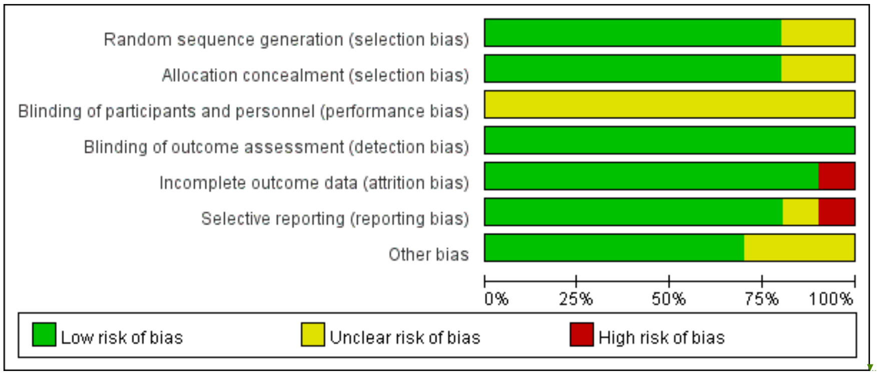

Supplemental Figure 4. Funnel plots depicts publication bias (A) All-cause mortality (B) Dialysis dependence (C) Free of dialysis

(A)

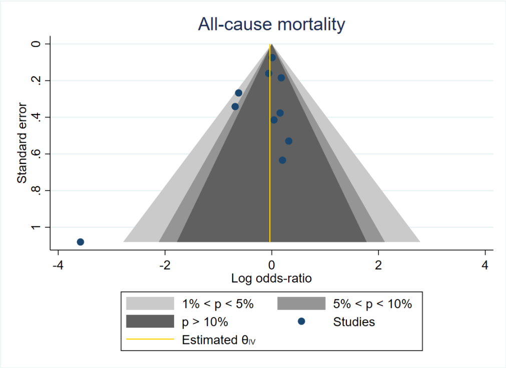

(B)

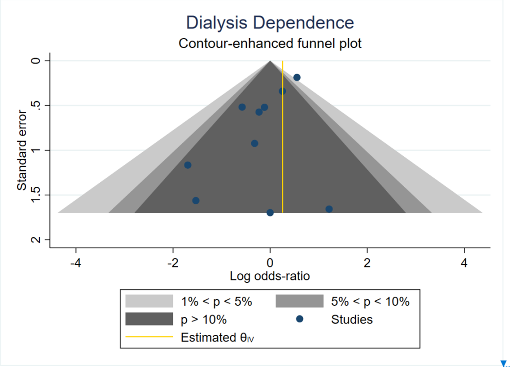

(C)

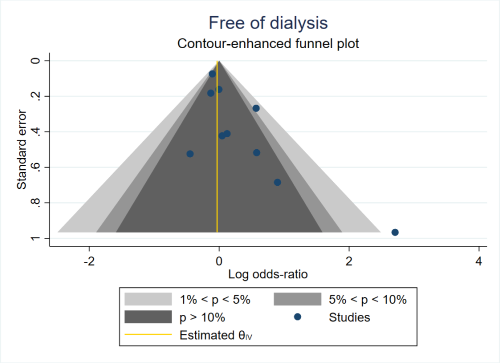

已刪除: 3

已刪除: P

已刪除: ↵

已刪除: ↵

已刪除: ↓

已刪除: ↵

Supplemental Figure 5. Forest plots for all-cause mortality comparing accelerated versus standard initiation of RRT among RCTs by random effects model

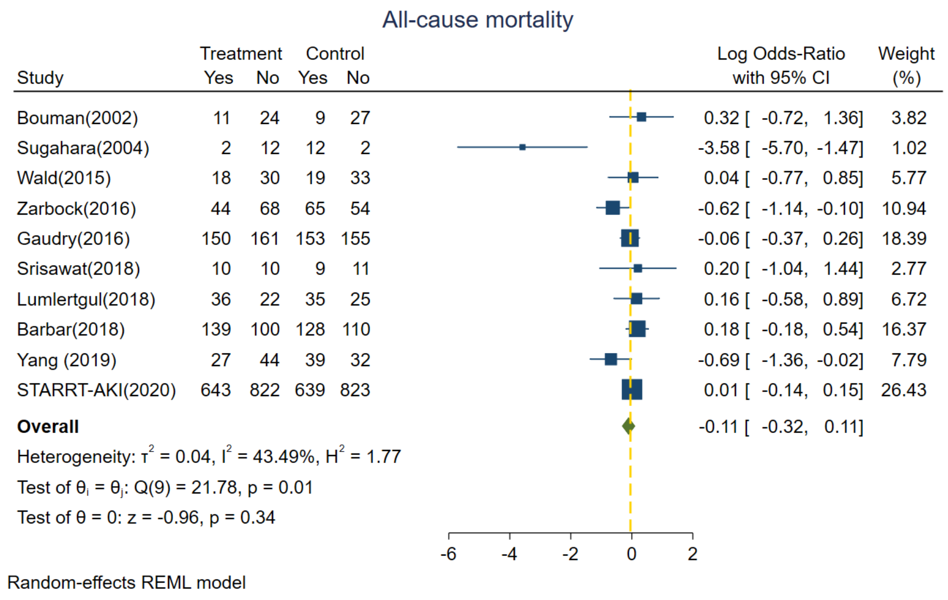

Abbreviations: CI: confidence interval; RCT: randomized controlled trials; RRT, renal replacement therapy.

已删除: in
已删除: .
已删除: ↵
已删除: ↵

Supplemental Figure 6 Forest plots for 28 and 90 days mortality comparing accelerated versus standard initiation of RRT among RCTs

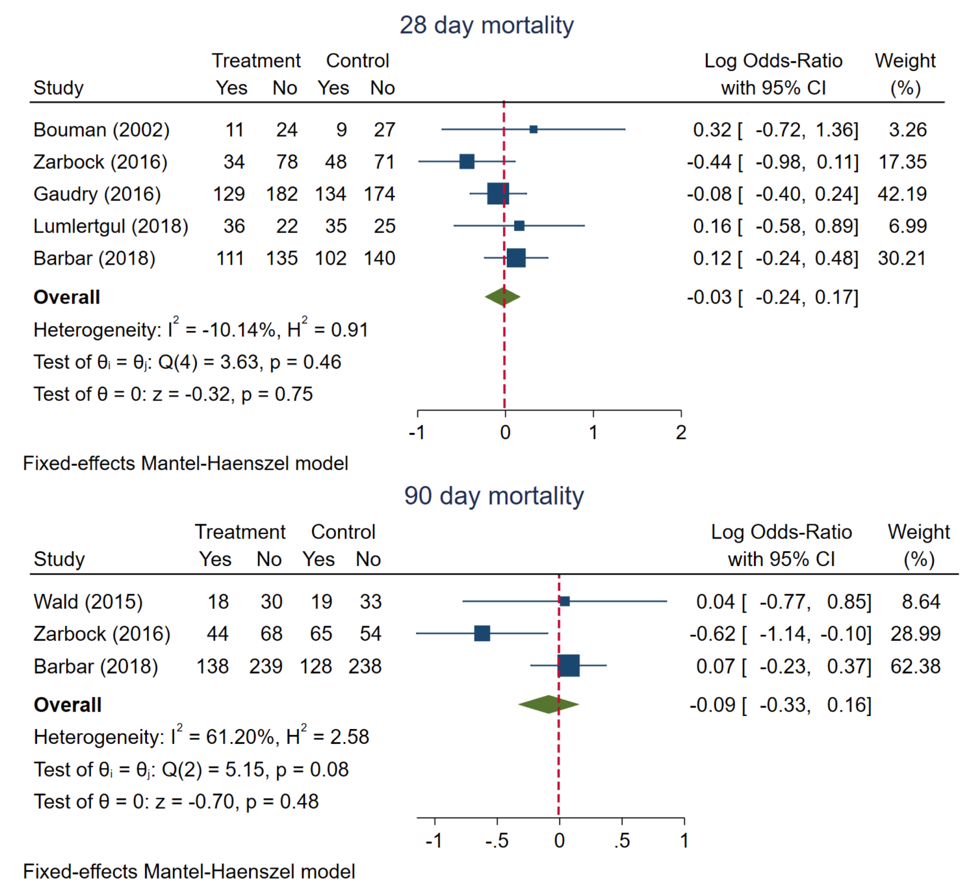

Abbreviations: CI: confidence interval; RCT: randomized controlled trials; RRT, renal replacement therapy.

已刪除: ↵

已刪除: **5**

已刪除: ↵
分頁符號

Supplemental Figure 7. Sensitivity analysis of subgroups for dialysis dependence comparing accelerated versus standard initiation of RRT among RCTs

已刪除: ↵

已刪除: .

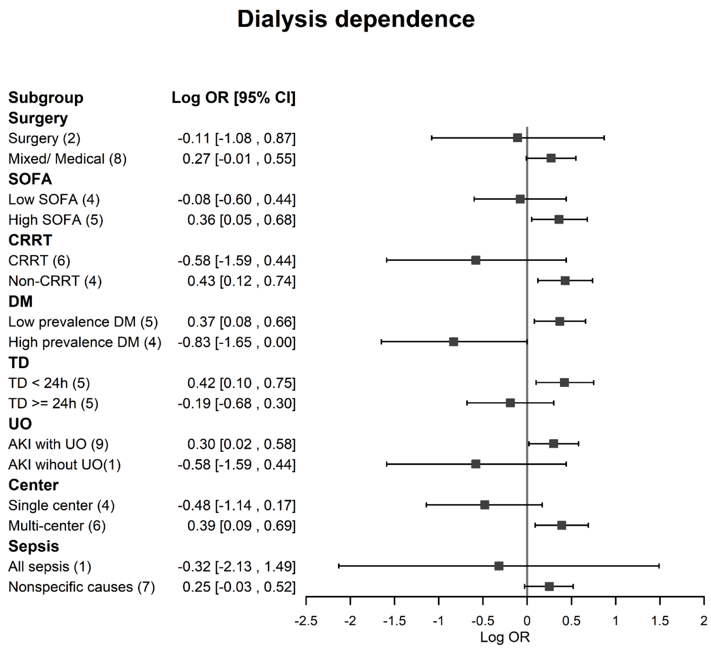

Abbreviations: CRRT, Continuous Renal. Replacement Therapy; TD, time to dialysis discrepancy, RCT: randomized controlled trials; RRT, renal replacement therapy; SOFA, Sequential Organ Failure Assessment; UO: urine output.

已刪除: <物件>↵

已設定格式: 字型: 非粗體

格式化: 行距: 2 倍行高

已刪除: ↵

已刪除: ↵

Supplemental Figure 8. Forest plots for dialysis dependence comparing accelerated versus standard initiation of RRT among RCTs in random effects model

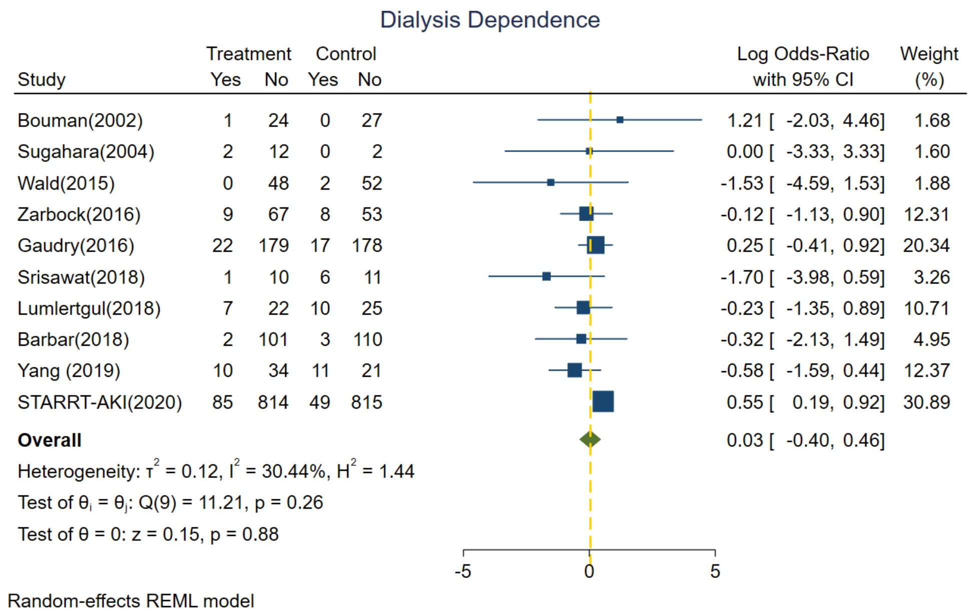

Abbreviations: CI: confidence interval; RCT: randomized controlled trials; RRT, renal replacement therapy.

已刪除: 6
已刪除: .
已刪除: ↵

Supplemental Figure 9. Forest plots for being free of dialysis comparing accelerated versus standard initiation of RRT among RCTs in random effects model

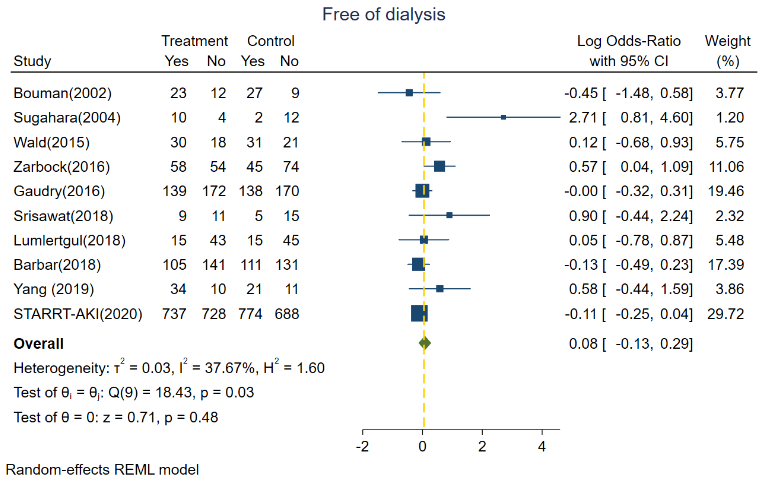

Abbreviations: CI: confidence interval; RCT: randomized controlled trials; RRT, renal replacement therapy.

Supplemental Figure 10. Hazzard ratio for all-cause mortality comparing accelerated versus standard initiation of RRT*

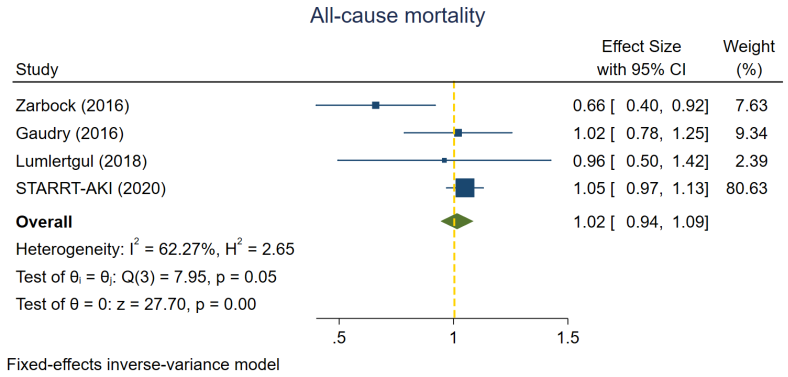

Abbreviations: CI: confidence interval; RCT: randomized controlled trials; RRT, renal replacement therapy.

* Included studies that provided hazard ratios of all-cause mortality.

已刪除: ↵ ↵ ↵ ↵
已刪除: ↵
已刪除: 7
已刪除: .
已刪除: ↵
格式化: 行距: 2 倍行高
已刪除: 8
已刪除: ↵ ↵
已刪除: ↵
格式化: 左右對齊, 行距: 固定行高 24 點
已刪除: CI: confidence interval.
已設定格式: 字型: (中文) Times New Roman
格式化: 左右對齊
已設定格式: 字型: (英文)Times New Roman
已設定格式: 字型: (英文)Times New Roman
已刪除: included studies provdes Hazrd ratio of all-cause mortality
已刪除:
已刪除: ---分頁符號---

Supplemental Figure 11. Forest plots for all-cause mortality comparing accelerated versus standard initiation of RRT (excluded Gaudry and Yang's study), grouped by surgery unit and CRRT modality

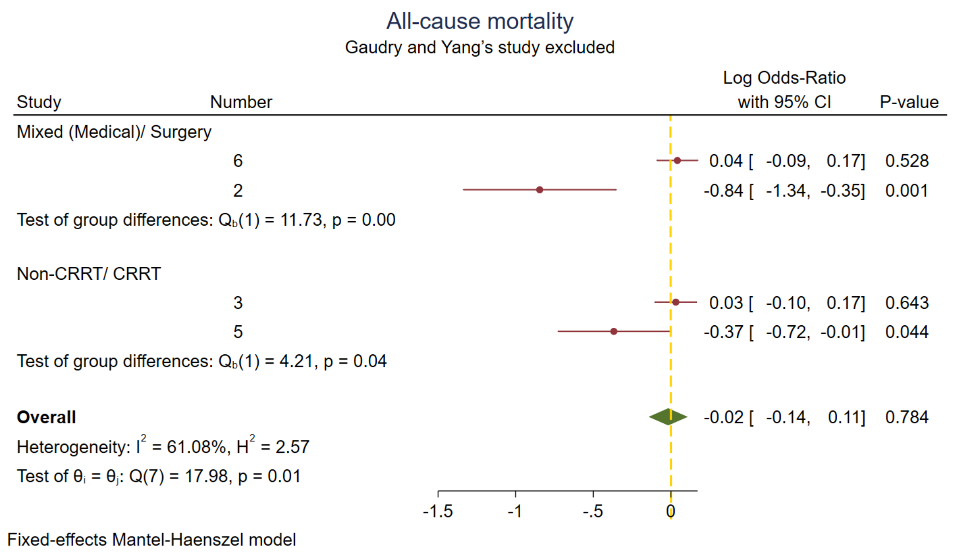

已删除:

Abbreviations: CI: confidence interval; RCT: randomized controlled trials; RRT, renal replacement therapy; SCI: Science Citation Index.

已删除:

Supplemental Figure 12. Forest plots for dialysis dependence comparing accelerated versus standard initiation of RRT (excluded Gaudry and Yang's study), grouped by surgery unit and CRRT modality

已刪除:

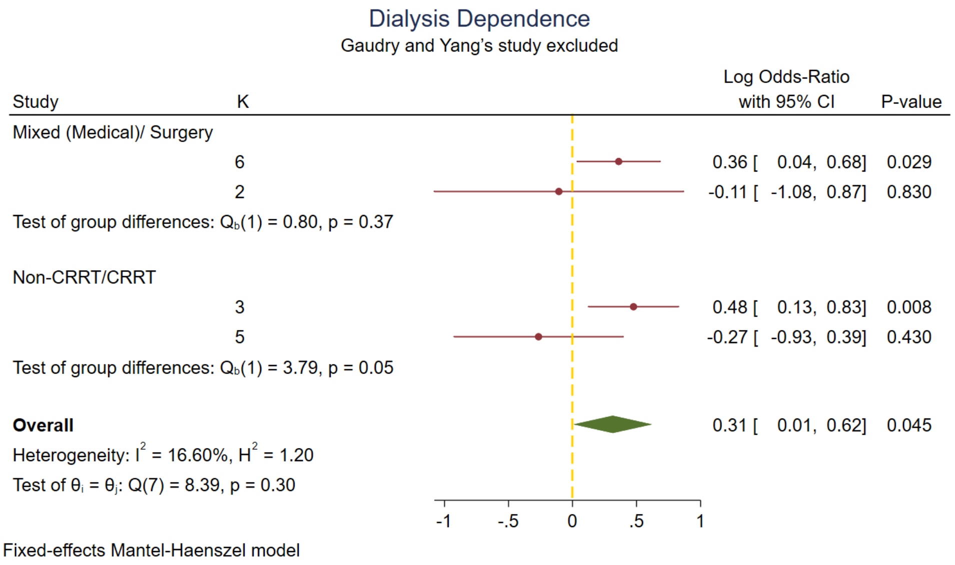

Abbreviations: CI: confidence interval; RCT: randomized controlled trials; RRT, renal replacement therapy; SCI: Science Citation Index.

Supplemental Figure 13 Forest plots for being free of dialysis comparing accelerated versus standard initiation of RRT (excluded Gaudry, and Yang's study), grouped by surgery unit and CRRT modality

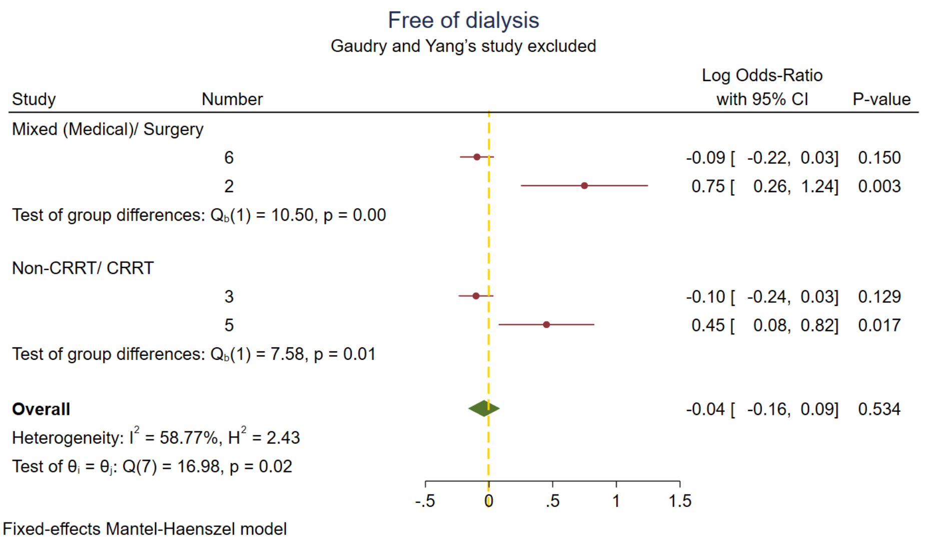

Abbreviations: CI: confidence interval; RCT: randomized controlled trials; RRT, renal replacement therapy; SCI: Science Citation Index.

已删除:

已删除: 1

已删除:

5. Summary of contextual factor data

In our study, 10 RCTs with 4753 participants were included in this meta-analysis. In Sugahara et al. (2004), 28 patients were included and early initiation of dialysis improved the survival rate with AKI following cardiac surgery (p<0.01). Zarbock et al. (2016, ELAIN study) included 231 critically ill patients with AKI and showed early initiation of RRT reduced 90-day mortality (p=0.03), RRT duration (p=0.04), and hospital stay (p<0.001). Yang et al. (2019) included 142 septic patients with AKI and showed lower 28-day mortality (p<0.05) and higher renal recovery rate (p<0.05) in early CRRT group.

Bouman et al. (2002) included 106 critically ill patients with oliguria and showed that early initiation or higher ultrafiltration volume hemofiltration did not improve the recovery of renal function and nor 28-day survival rate. Wald et al. (2015) was a multicenter, open-label study which included 101 critically ill AKI patients, however, the mortality, dialysis dependence and duration of ICU and hospital stay were not significantly different between accelerated and standard RRT. Gaudry et al. (2016, AKIKI Study) is a multicenter study including 620 critically ill AKI patients and showed no significant difference of an early and delayed strategy in mortality (p=0.79). In Barbar et al. (2018, IDEAL-ICU study), 488 septic shock with AKI patients were included and 90-day mortality showed no significant difference in 90-day mortality (p=0.38) between early and delayed RRT strategy. Lumlertgul et al. (2018, FST trial) included 118 FST-nonresponsive patients and showed no difference in 28-day morality (p=0.68) and RRT dependence at day 28 (p=0.77). In Srisawat et al. (2018), 40 patients with high pNGAL were included and there was not significant difference in 28-day morality (p=0.72) and dialysis dependence (p=0.062) between early and standard RRT. The STARRT-AKI et al. (2020) is a multinational trial included critically ill AKI patients, and accelerated initiation of RRT did not decrease 90-day mortality compared to standard RRT groups (p=0.92).

From most of the references above, the accelerated initiation of RRT in AKI patients did not improve the mortality and other outcomes. However, to protocolize the optimal timing of RRT for all critically ill AKI patients may be difficult in the personalized medicine era. And the negative result on the primary outcomes might be hidden among a higher level of heterogeneity in terms of disease progression, that could not be predicted by AKI staging at the time of inclusion.

已刪除: ↵ ↵ ↵ ↵

已刪除: ↵

已刪除: 3

已刪除: 5

已刪除: ,366

已刪除: In Pursnani et al. (1997), 35 cases were included and early hemodialysis had lower mortality (22.2% vs 29.4%) and shorter hospital stay. Durmaz et al. (2003) included 44 patients with creatinine >2.5 mg/dL not requiring dialysis receiving coronary artery bypass surgery with cardiopulmonary bypass and showed prophylactic hemodialysis decreasing mortality (p=0.048), AKI development (p=0.023) and the length of ICU and hospital stay (p=0.005 and p=0.023).

已設定格式: 字型: (英文)Times New Roman

已刪除: .

已刪除: 5,229

已刪除: Koo et a. (2006) included 102 patients with sepsis or septic shock and early initiation of CVVH before AKI development may improve survival rate (p<0.001).

已刪除: dependent

已設定格式: 字型: (英文)Times New Roman, (中文) Times New Roman, 不加底線, 字型色彩: 自動

已刪除: did not have significant difference

已刪除: Combes et al. (2015, HEROICS Study) was a multicenter study with eligible 112 patients receiving high volume hemofiltration, and early initiation did not improve 30-day mortality and other outcomes compared to standard strategy.

Of note, our data revealed accelerated initiation of RRT may be beneficial in critically ill AKI patients regarding survival and free of dialysis in surgical ICU settings or those receiving CRRT modality. However, there are several limitations in this study, including the information regarding other factors of mortality, the standardized dialysis modality and dose delivered, the negative results which were unpublished and the diversity of accelerated RRT definition, that might cause bias of the analysis in our study. Most importantly, the definition of accelerated RRT varied in these studies and had influenced pooled effect estimates. Therefore, to investigate different etiologies of AKI affects outcomes of RRT timing (accelerated versus standard) or to evaluate CRRT efficacy fits in various underlying causes of AKI in critically ill patients can be the issues incorporating in future RCTs design to evaluate the optimal timing and modalities of RRI in critically ill AKI patients.

In conclusion, critically ill AKI patients benefit from accelerated RRT regarding all-cause mortality and free of dialysis if they were surgical ICU patients or receiving CRRT treatment. However, the risk of dialysis dependence was increased in accelerated RRT group, when those AKI patients were non-CRRT and of high disease severity groups. All the literatures in this meta-analysis were highly heterogeneous and potentially subject to low biases.

已删除: ↵

已删除: ↵

已删除: ↵

6. PROSPERO protocol registration.

NIHR | National Institute for Health Research

PROSPERO
International prospective register of systematic reviews

Timing of initiation of renal replacement therapy in acute kidney injury in critically ill patient- updated systematic review and meta-analysis

Yingying Chen, VinCent Wu

To enable PROSPERO to focus on COVID-19 registrations during the 2020 pandemic, this registration record was automatically published exactly as submitted. The PROSPERO team has not checked eligibility.

Citation

Yingying Chen, VinCent Wu. Timing of initiation of renal replacement therapy in acute kidney injury in critically ill patient- updated systematic review and meta-analysis. PROSPERO 2020 CRD42020201466 Available from:
https://www.crd.york.ac.uk/prospero/display_record.php?ID=CRD42020201466

Review question

Dose early initiation of renal replacement therapy improve the outcome including survival and renal recovery in critically ill patients with acute kidney injury?

Searches

Search database: PubMed, Embase, Cochrane.

Search date: since January 1st, 1997 to July 20th, 2020.

Restrict to English and human study, the publication period as the search date.

The search will be re-run prior to the final analysis.

The unpublished studies will be sought.

Types of study to be included

We will include randomized control trials only to access the impact of the early initiation of renal replacement therapy.

Condition or domain being studied

Acute kidney injury, renal replacement therapy, critical illness

Participants/population

Inclusion criteria: adult with acute kidney injury and admitted intensive care units

Exclusion criteria: adolescents (under 18 years of age)

Intervention(s), exposure(s)

Intervention:early initiation of renal replacement therapy defined as patients received renal replacement therapy in accordance with stage, time duration, or blood exam when acute kidney injury occurred.

Comparator(s)/control

Control: when acute kidney injury occurred, in compare with early definition, patients did not received renal replacement therapy immediately.

Main outcome(s)

Mortality, renal replacement therapy dependence

* Measures of effect

relative risks, hazard ratio, and odds ratios

Additional outcome(s)

free from dialysis

* Measures of effect

relative risks, hazard ratio and odds ratios

Data extraction (selection and coding)

Data will be extracted by two researchers reviewed literature independently according to the Cochrane guidelines.A third investigator will be available when there were any disagreements regarding to data extraction and/or quality assessment. The quality of these RCTs was assessed by Review Manager 5.3 (Oxford, UK).

Data extraction:We extract data including leading author, year of publication, study location(surgical/medical/mixed ICU), patient number and cohort size, criteria used for AKI diagnosis, definition of "early" or "late" initiation, inclusion and exclusion criteria, patient population, RRT modality, presence of sepsis, disease severity score, and the proportions of patients on mechanical ventilation. We obtain the information from published articles.

We will try to contact the original author to obtain the missing data or additional details.

Risk of bias (quality) assessment

We assess the characteristics including random sequence generation, allocation concealment, blinding of

participants and researchers, blinding of outcome assessment, incomplete outcome data, and selective reporting.

The assessment will be done at study or outcome level.

We also assess the internal validity.

Two reviewer will take the responsibility to assess the quality.

A third reviewer will be available if there were any disagreements between the two reviewers.

Strategy for data synthesis

We search for the data in accordance with the key words and limit to RCT study, and the data is including the hazard ratio, odds ratio or risk ratio of the mortality or renal replacement therapy.

Analysis of subgroups or subsets

The subgroup we analysis will include ICU type, RRT modality, disease severity, time duration, and AKI stage.

Contact details for further information

Yingying Chen
akochen45@hotmail.com

Organisational affiliation of the review

Mackay Memorial Hospital
https://post.mmh.org.tw/english/

Review team members and their organisational affiliations

Dr Yingying Chen. Division of Nephrology, Department of Internal Medicine, Memorial Hospital
VinCent Wu. Division of Nephrology, Department of Internal Medicine, National Taiwan University Hospital

Type and method of review

Systematic review

Anticipated or actual start date
16 July 2020

Anticipated completion date
12 September 2020

Funding sources/sponsors
We do not receive any funding or have any sponsor.

Conflicts of interest

Language
English

Country
Taiwan

Stage of review
Review Ongoing

Subject index terms status
Subject indexing assigned by CRD

Subject index terms
MeSH headings have not been applied to this record

Date of registration in PROSPERO
07 September 2020

Date of first submission
07 August 2020

Stage of review at time of this submission
The review has not started

Stage	Started	Completed
Preliminary searches	No	No
Piloting of the study selection process	No	No
Formal screening of search results against eligibility criteria	No	No
Data extraction	No	No
Risk of bias (quality) assessment	No	No
Data analysis	No	No

The record owner confirms that the information they have supplied for this submission is accurate and complete and they understand that deliberate provision of inaccurate information or omission of data may be construed as scientific misconduct.

The record owner confirms that they will update the status of the review when it is completed and will add publication details in due course.

NIHR | National Institute for Health Research

PROSPERO
International prospective register of systematic reviews

Versions

07 September 2020

PROSPERO
This information has been provided by the named contact for this review. CRD has accepted this information in good faith and registered the review in PROSPERO. The registrant confirms that information supplied for this submission is accurate and complete. CRD bears no responsibility or liability for the content of this registration record, any associated files or external websites.

格式化: 行距: 固定行高 12 點
已刪除:

7. The GRADE results

Author(s): Heng-Chih Pan, Ying-Ying Chen, I-Jung Tsai, Chih-Chung Shiao ,Tao-Min Huang, Chieh-Kai Chan, Hung-Wei Liao, Tai-Shuan Lai, Vin-Cent Wu, MD, Yung-Ming Chen

Question: Accelerated Initiation of RRT compared to standard Initiation of RRT for critically-ill patients with acute kidney injury

Setting: Any

Bibliography:

Certainty assessment							№ of patients		Effect		Certainty	Importance
**№ of studies**	**Study design**	**Risk of bias**	**Inconsistency**	**Indirectness**	**Imprecision**	**Other considerations**	**accelerated Initiation of RRT**	**standard Initiation of RRT**	**Relative (95% CI)**	**Absolute (95% CI)**		
**All cause mortality**												
10	randomised trials	not serious	serious [a]	not serious	not serious	none	1080/2373 (45.5%)	1108/2380 (46.6%)	**Log OR -0.04** (-0.16 to 0.07)	**-- per 1,000** (from -- to --)	⨁⨁⨁◯ MODERATE	CRITICAL
**RRT dependence**												
10	randomised trials	not serious	not serious	not serious	not serious	none	139/2373 (5.9%)	106/2380 (4.5%)	**Log OR 0.24** (-0.03 to 0.51)	**-- per 1,000** (from -- to --)	⨁⨁⨁⨁ HIGH	CRITICAL
**Free of dialysis**												
10	randomised trials	not serious	serious [a]	not serious	not serious	none	1160/2373 (48.9%)	1169/2380 (49.1%)	**Log OR -0.03** (-0.14 to 0.09)	**-- per 1,000** (from -- to --)	⨁⨁⨁◯ MODERATE	CRITICAL

CI: Confidence interval

Explanations

a. There was a high I2 value

已刪除: ↵

已設定格式: 字型: (英文)Times New Roman, 粗體, 字型色彩: 黑色

格式化: 清單段落

格式化: 行距: 多行 1.15 li

8. Flow chart showing Search strategy for studies in China.

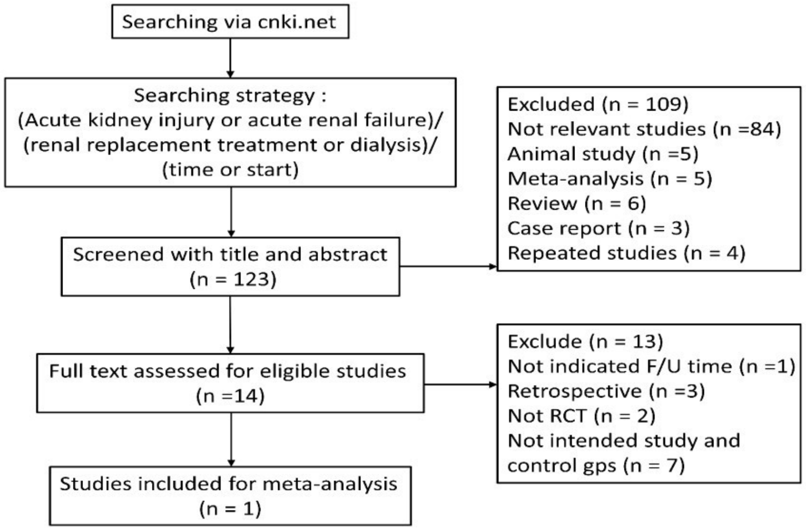

第 6 頁: [1] 已刪除 Apple 2020/11/6 11:58:00 PM

第 6 頁: [2] 已刪除 潘恆之 2020/11/14 4:24:00 PM

第 6 頁: [3] 已刪除 Apple 2020/11/6 11:58:00 PM

第 6 頁: [4] 已刪除 潘恆之 2020/11/9 5:04:00 PM
